# Considering the Influence of Coronary Motion on Artery-Specific Biomechanics Using Fluid–Structure Interaction Simulation

**DOI:** 10.1007/s10439-023-03214-0

**Published:** 2023-07-12

**Authors:** Nicholas A. T. Fogell, Miten Patel, Pan Yang, Roosje M. Ruis, David B. Garcia, Jarka Naser, Fotios Savvopoulos, Clint Davies Taylor, Anouk L. Post, Ryan M. Pedrigi, Ranil de Silva, Rob Krams

**Affiliations:** 1 National Heart and Lung Institute, Imperial College London, Guy Scadding Building, Cale Street, London, SW3 6LY UK; 2 Simulia, Dassault Systemes UK Ltd, Knutsford, UK; 3 Mechanical & Materials Engineering, University of Nebraska-Lincoln, Lincoln, USA; 4 Amsterdam UMC, Department of Biomedical Engineering and Physics, University of Amsterdam, Amsterdam, The Netherlands; 5 School for Material Sciences and Engineering, Queen Mary University, London, UK

**Keywords:** Shear stress, Endothelial strain, Coronary bending, Computational fluid dynamics, Coronary biomechanics

## Abstract

The endothelium in the coronary arteries is subject to wall shear stress and vessel wall strain, which influences the biology of the arterial wall. This study presents vessel-specific fluid–structure interaction (FSI) models of three coronary arteries, using directly measured experimental geometries and boundary conditions. FSI models are used to provide a more physiologically complete representation of vessel biomechanics, and have been extended to include coronary bending to investigate its effect on shear and strain. FSI both without- and with-bending resulted in significant changes in all computed shear stress metrics compared to CFD (*p* = 0.0001). Inclusion of bending within the FSI model produced highly significant changes in Time Averaged Wall Shear Stress (TAWSS) + 9.8% LAD, + 8.8% LCx, − 2.0% RCA; Oscillatory Shear Index (OSI) + 208% LAD, 0% LCx, + 2600% RCA; and transverse wall Shear Stress (tSS) + 180% LAD, + 150% LCx and + 200% RCA (all *p* < 0.0001). Vessel wall strain was homogenous in all directions without-bending but became highly anisotropic under bending. Changes in median cyclic strain magnitude were seen for all three vessels in every direction. Changes shown in the magnitude and distribution of shear stress and wall strain suggest that bending should be considered on a vessel-specific basis in analyses of coronary artery biomechanics.

**Supplementary Information:**

The online version contains supplementary material available at 10.1007/s10439-023-03214-0.

## Introduction

In the arterial circulation, endothelial cells (ECs) are exposed to time-varying shear stress, resulting from the in-plane frictional force generated by changing blood flow during the cardiac cycle. Coronary arteries undergo motion during the cardiac cycle, resulting in exposure of ECs to cyclical wall stress and strain, which can influence their biological behaviour [11, 21, 25, 28]. Extensive clinical and pre-clinical work has demonstrated that disturbed shear stress results in altered EC morphology and biology [12, 28] that is pro-atherogenic. In contrast, disturbed mechanical strain has been less studied, despite it also being a potent driver of pro-inflammatory and pro-atherogenic pathways in ECs [6, 8, 20–22, 29]. Given that disturbances in both biomechanical factors directly alter EC biology, the ability to assess both the fluid and solid biomechanical domains within the coronary circulation in vivo may have potential to advance investigation of mechanisms underlying coronary atherogenesis.

Computational approaches enable assessment of the biomechanical environment within coronary arteries. Most studies consider the fluid and solid domains independently using computational fluid dynamics (CFD) and finite element modelling (FEM) [2], respectively. Fluid–structure interaction (FSI) modelling seeks to reconcile this limitation by modelling both the fluid and solid domains simultaneously, therefore providing a more complete physiological description of the shear stress and wall strain to which ECs are exposed to in vivo.

The influence of cardiac motion on coronary haemodynamics has been the subject of several studies. Computational analysis of a simplified straight vessel under cyclic axial displacement revealed a significant effect on wall shear and Oscillatory Shear Index (OSI) [26]. Schilt et al. [38] demonstrated a clear variation in flow velocity distribution from inner to outer curvature in an experimental study on a curved cylinder undergoing combined axial and perpendicular planar cyclic motion, noting the effect was a dynamic phenomenon related to the constant change in curvature. CFD simulation of a curved cylinder displaced at a frequency of 1 Hz showed only small changes in the distribution of wall shear rate compared to a static model [37], however a displacement frequency of 5 Hz indicated an obvious difference between inner wall shear rates for the static and dynamic case [25]. Ramaswamy et al. [36] and Zeng et al. [53] performed CFD analyses on a reconstructed LAD and RCA respectively, providing more physiological representative studies, which also revealed an influence of cyclic bending on WSS distributions. More recently, studies based on FSI simulation methods have been presented, incorporating the distensibility of the vessel wall, such as that of Wang et al. [48] who found influence of cyclic bending on time averaged WSS and OSI as a function of heart rate.

FSI analyses also include the mechanical effect on the vessel wall [9], with studies on stenotic coronary arteries [44, 49] showing bending to cause considerable variations in strain in plaques, of up to 134% [52]. Thus, the literature strongly suggests the inclusion of cyclic bending for a complete representation of coronary biomechanics, and also highlights the complexities of recreating it accurately, such as the phase difference between blood flow and motion [33, 35], the unique non-planar displacement [38], and the non-sinusoidal nature of the motion [25]. Subject-specific studies with coherent, co-registered boundary conditions provide a means to address these more comprehensively [42].

In this report, we develop a framework for in vivo coronary artery-specific FSI modelling, which incorporates the influence of coronary motion, using experimentally acquired arterial geometries and vessel-specific boundary conditions. We verify this model experimentally and show that this physiologically appropriate FSI model results in significant alterations in estimates of shear stress metrics known to be associated with coronary atherogenesis, as well as strain.

## Materials and Methods

### Animal Procedures

Experimental procedures are described in Supplementary Methods.

### 3D Artery Reconstruction

Coronary artery geometries were reconstructed as previously reported [30, 34, 40], detailed in Supplementary Methods.

### Fluid–Structure Interaction and Computational Fluid Dynamic Modelling

#### Fluid Domain

Blood flow simulation (fluid domain) in FSI and CFD models was implemented using Abaqus\CFD 6.14 [39]. Blood was modelled as a laminar incompressible non-Newtonian fluid, with a density of 1050 kg/m^3^. Its dynamic viscosity was modelled by the Carreau model [17], with a viscosity range of 3.45 × 10^−6^ mPa to 5.6 × 10^−5^ mPa, time constant *λ*_t_ = 3.313, flow behaviour index *n* = 0.3568, and parameter *a* = 2. The inner wall of the artery was treated as a no-slip boundary.

#### Solid Domain

For FSI simulations, the solid domain (Supplementary Fig. 1) consists of the artery wall and its surrounding perivascular support material. This provides mechanical support, preventing unrealistic movement and oscillation of the artery. Solid domain simulations were performed with Abaqus/Standard 6.14 [39]. Co-simulation of Abaqus/CFD and Abaqus/Standard was used for FSI analyses, where the fluid–solid interface was the inner surface of the artery wall.

The artery wall was modelled as a hyperelastic material using an isotropic three-term Ogden constitutive model [13], with a strain energy function given by

1 $$W={\sum }_{i=1}^{N}\frac{2{\mu }_{i}}{{{\alpha }_{i}}^{2}}\left({{\lambda }_{1}}^{\alpha i}+{{\lambda }_{2}}^{\alpha i}+{{\lambda }_{3}}^{\alpha i}- 3\right),$$

where *λ*_1_, *λ*_2_ and *λ*_3_ are the deviatoric stretches and the coefficients µ_i_ and α_i_ are material parameters; *µ*_1_ = 6.8991 kPa, *α*_1_ = 8.5782, *µ*_2_ = 10.0284 kPa, *α*_2_ = 0.0003, *µ*_3_ = 3.9691 kPa, *α*_3_ = 8.5782, *D*_1_ = 9.57 × 10^−4^. The ratio of initial bulk modulus to initial shear modulus, ${\mu }_{0}/{K}_{0}$, was 100, equating to a Poisson’s ratio of $\nu =0.495.$ Parameter *D*_1_ was calculated accordingly via

2a,b $${\mu }_{0}=\sum_{i=1}^{N}{\mu }_{i},\;{K}_{0}=\frac{2}{{D}_{1}}$$

Ogden material coefficients *µ*_*i*,_ *α*_*i*_ were derived from non-linear regression fitting of the stress–strain output obtained by Wang et al. [47] via experimental inflation testing of porcine coronary arteries. The perivascular support material was modelled as a linear elastic material with a Poisson’s ratio of 0.05 and Young’s modulus of 10 kPa, selected to ensure artery wall expansion was not restricted.

For FSI simulations without bending, the inlet and outlet faces of the artery wall were constrained in the axial and circumferential directions, but free to move in the radial direction, Supplementary Fig. 2. The outer surface of the perivascular material was constrained in all directions, Supplementary Fig. 3. Mesh convergence tests demonstrated less than 0.2% difference in mean fluid velocity and less than 1.5% variation in strain at the chosen mesh resolution. Details on meshing of the fluid and solid domains are provided in Supplementary Methods, alongside a DOI link to a repository containing input files for the LAD simulations.

#### Physiological Artery-Specific Boundary Conditions

##### Fluid Domain

**Fig. 1:**
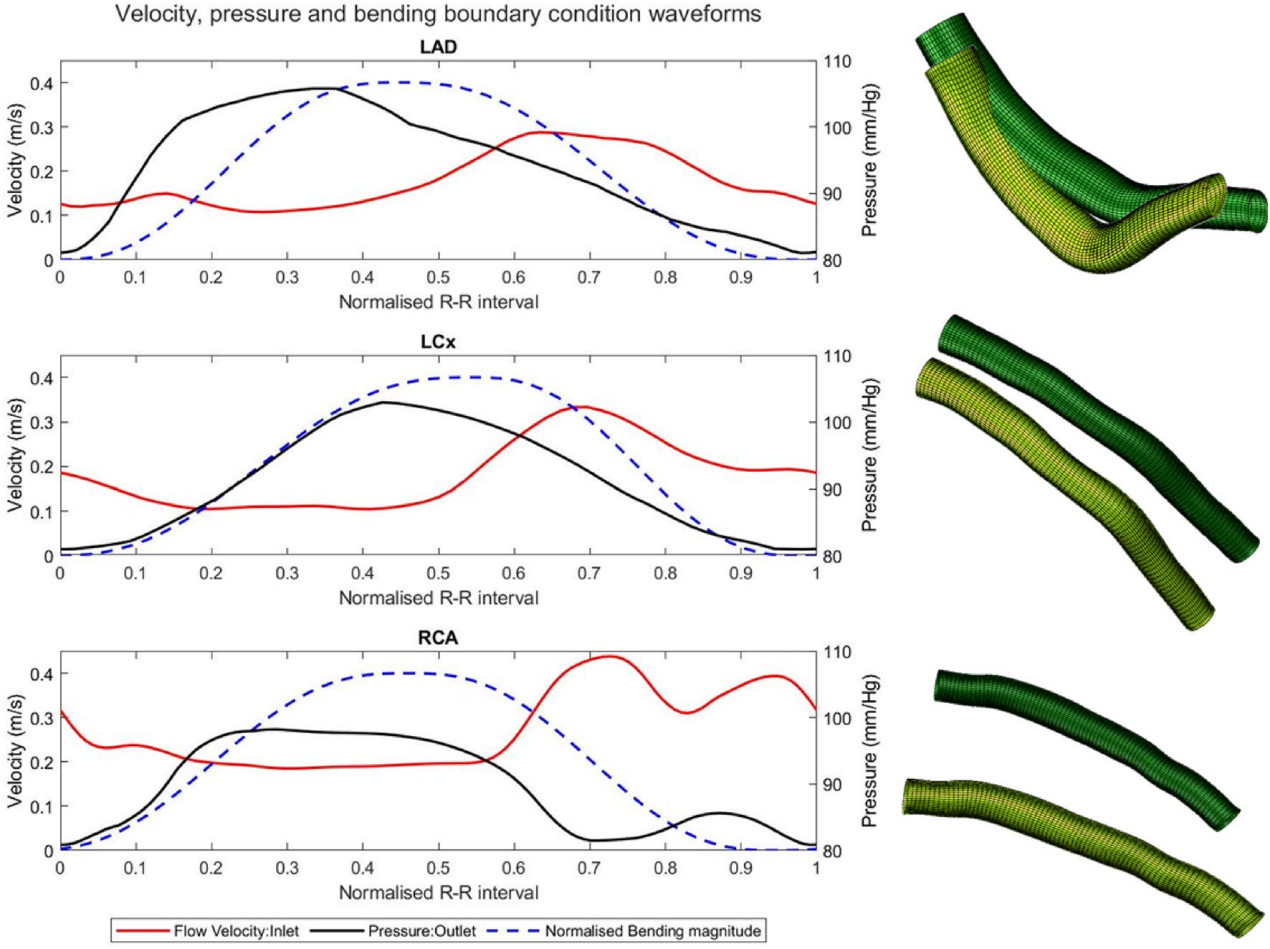
Summary of applied fluid-domain boundary conditions. Plots showing time-varying waveforms of flow velocity (vessel inlet), pressure (vessel outlet) and bending amplitude, for **a** the LAD, **b** the LCx, and **c** the RCA. Waveforms are normalised to the R-R interval for comparison, but cardiac cycle time periods vary between vessels due to difference in subject heart rates at the time of measurement. Accompanying images show the diastolic (dark green) and systolic (light green) configurations of the **d** LAD, **e** LCx and **f** RCA, providing qualitative description of the bending displacements applied to each vessel. The inlet extension part of the model is not shown

Directly measured artery-specific boundary conditions were applied to the fluid domain. Blood flow velocity and pressure were measured proximally and distally to provide a time-varying waveform. Raw data (velocity at the inlet and pressure at the outlet) were smoothed to create a cyclic waveform applied to both the FSI and CFD simulations; (Fig. 1). The inlet velocity was applied as a parabolic profile using the measured velocity as the maximum. An inlet extension of 1.5 times the inlet diameter was added to allow for fully developed flow.

##### Solid Domain

The thickness of the artery wall within the FSI model was measured in vivo for each artery over multiple OCT frames (LAD = 250 µm; LCx and RCA = 190 µm), and applied as a uniform wall thickness along the entire length of the model.

#### Artery-Specific Coronary Bending Model

Incorporation of coronary bending into the FSI model was achieved by prescribing a displacement to nodes situated on the outer layer of the artery wall. The bending displacements were calculated individually for each artery, using the centreline of the un-instrumented artery at systole and diastole extracted from 3D-QCA reconstructions (Supplementary Fig. 4). The difference in position between the diastolic and systolic centreline coordinates was calculated, providing a set of 3D displacement vectors for each point on the centreline. These displacement vectors were assigned to the nearest circumferential set of nodes on the mesh, allowing the applied displacement to vary axially.

The prescribed displacement was applied to a section of the artery wall covering approximately 30% of the circumference, located on the inner curvature where the artery would be embedded in the myocardium (Supplementary Fig. 5). This was derived from selected pathology sections of the studied coronary arteries, providing a realistic bending loading whilst limiting the effect on vessel wall distension. Nodes lying on the same circumferential set of elements share the same applied displacement.

Bending displacements (derived from 3D-QCA) were applied using an amplitude function varying between 0 and 1; maximum displacement was applied at systole, and zero displacement at diastole. This amplitude was synchronised with the haemodynamical boundary conditions using the electrocardiogram associated with the 3D-QCA and combomap measurements. This ensures the prescribed cyclical movement between diastolic and systolic configurations is aligned in time with the applied flow velocity and pressure waveforms. A smoothed asymmetrical sawtooth waveform is used for the bending amplitude (Fig. 1) to avoid applying a large impulse to the model when the artery reverses direction at end-systole and end-diastole.

Both the inlet and outlet of the artery translate under the bending load (Fig. 1). A rigid-body plane with sliding contact is included in the model at both faces to permit radial dilatation whilst locally constraining nodes axially to remain in-plane, thereby maintaining periodic boundary conditions. Bending displacement was also applied to the full circumference of the outer surface of the perivascular material (Supplementary Fig. 3), using the same amplitude and axial-position-based displacement vectors as for the vessel wall.

#### Inclusion of Physiological Pre-stress

Coronary artery geometries derived from experimentally acquired in vivo source data were used, but as this does not represent a zero-load configuration, in vivo stresses must be accounted for [10]. The residual stress associated with the opening angle has not been included, but those owing to the intrinsic axial stretch loading and the diastolic pressure state were calculated, and applied as initial conditions to the FSI simulations.

##### Axial Loading

The backward incremental (BI) method [3, 41] was used to calculate stresses owing to the axial stretch using Abaqus\Standard. The axial load applied to the artery inlet and outlet is incrementally increased from 0 to 100 kPa in 10 steps and remains constant at 100 kPa thereafter. This pre-stress is equivalent to a physiological axial stretch ratio of ${\lambda }_{z}=1.3{-}1.4$ [47].

##### Diastolic Pressure Inflation Pre-stress

After computing the axial stretch loading, the force due to the diastolic blood-pressure was applied. As before, the BI method was used to increase the pressure to 80 mmHg in 12 steps using Abaqus/Standard [27, 41]. At the first step, the stresses from the axial stretch loading were applied as an initial condition. The inlet and outlet of the artery were constrained in the axial direction throughout to maintain axial stress, whilst free to move in the radial and circumferential directions. After completion of the pressurization procedure, the stresses within the artery represented the physiological in vivo state at diastole.

#### Shear and Strain Metric Post-Processing

Time Averaged Wall Shear Stress (TAWSS) was calculated at each element on the lumen wall and averaged over one cardiac cycle [45]. Transverse wall Shear Stress (tSS) and Oscillatory Shear Index (OSI) metrics were calculated as detailed in Supplementary Methods [24, 31, 32]. All strains are logarithmic and computed at the innermost layer of elements in the artery wall. Strains are presented as cyclic strain, which indicates maximum deformation from the diastolic state [27].

#### Estimation of Coronary Bending Metric

To quantify the amount of bending occurring in each vessel, the first derivative in space of the magnitude of the bending displacement was calculated along the length of each artery. This was used to assess the variation of bending along each artery and compare the degree of bending between arteries. Prior to calculating the derivative, the bending vectors were normalised to those applied at the inlet, to remove large rigid-body translations. A derivative of zero suggests equal bending displacement applied to successive sections of the artery length, i.e. a simple translation. Non-zero values represent differences between adjacent bending displacements, representing a local change in the artery centreline, and therefore, local deformation due to bending.

#### Statistics

Numerical data are expressed as median [interquartile range]. The distribution of the shear stress metrics for each modelling method were compared using a Kruskal–Wallis test with Bonferroni correction for multiple comparisons. Strain data were analysed with paired Wilcoxon Signed Rank tests. All *p* values are shown after Bonferroni correction with *p* < 0.05 considered statistically significant.

## Results

### FSI Model Verification

**Fig. 2:**
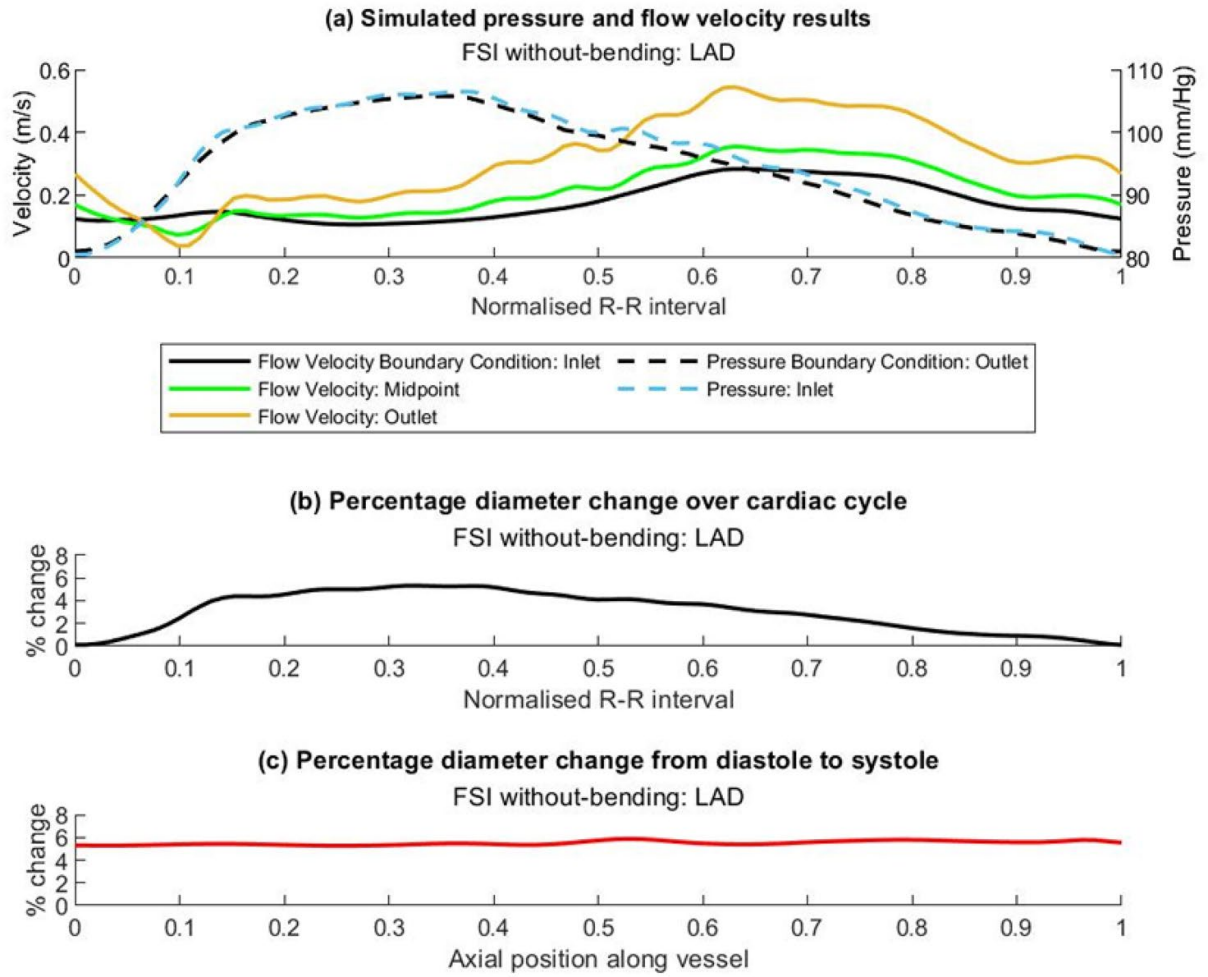
Results for fluid and solid domain of LAD FSI simulation without-bending, providing verification of physiologically valid results. **a** Plot showing flow velocity boundary condition applied at the vessel inlet, and pressure boundary condition applied at the outlet. Computed values of flow velocity at the vessel mid-point and outlet show increase in velocity as the vessel tapers. Computed value of pressure at the inlet confirms the presence of a pressure gradient. **b** Plot showing variation of computed mean vessel diameter with time, confirming distension of vessel wall in response to computed blood pressure forces. Values presented for a point located 25% along the length of the vessel. **c** Plot of percentage diameter change of vessel between diastole and systole along the length of the vessel. Mean dilation calculated over the proximal first 1/3 of the vessel of 5.3% compares well to in vivo measurement via IVUS of 4.5% ± 1%, measured in the proximal region of LAD

Flow velocity and pressure simulation results for the FSI without-bending simulation of the LAD are shown in Fig. 2a. Computed flow velocity values at the vessel mid-point and outlet show increased velocity as the vessel tapers. Comparison of computed inlet pressure to the applied outlet pressure confirms a pressure gradient along the vessel and a physiologically appropriate flow field. Figure 2b shows the computed distension of the vessel over the cardiac cycle, measured at one axial position, which is clearly seen to vary synchronously with pressure (Fig. 2a). Figure 2c shows results for the calculated maximum distension of the artery wall at all axial positions. The increase in mean systolic artery diameter over the proximal third of the LAD was calculated to be 5.3% which compares favourably with in vivo measurements of 4.5% ± 1% obtained in a similar location using intravascular ultrasound, and is also consistent with the observations of Weissman, Palacios and Weyman [50]. This supports the selected artery wall constitutive properties as providing a valid and realistic material response, and that the simulation configuration and applied boundary conditions produce physiological results in the fluid and solid domains for all simulations.

### Predicted Wall Shear Stress Metrics: FSI Without-Bending Compared with CFD

**Fig. 3:**
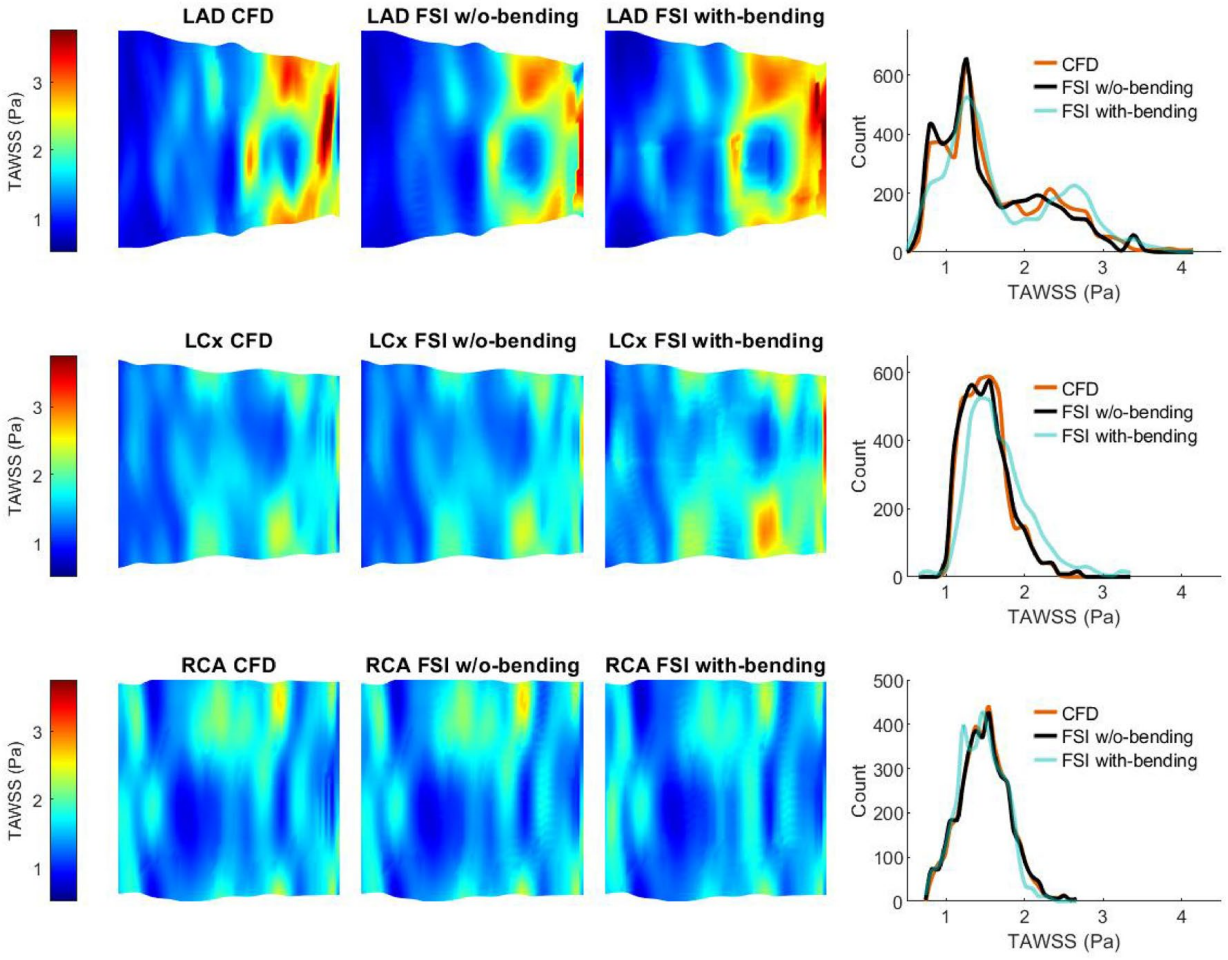
Results of Time Averaged Wall Shear Stress (TAWSS) for rigid-wall CFD, FSI without (w/o) bending, and FSI with-bending, for LAD, LCx and RCA. Maps of each artery are presented as opened and flattened, showing magnitude and location of TAWSS for flow from proximal (left) to distal (right). Histograms compare the CFD, FSI without-bending, and FSI with-bending TAWSS datasets for each vessel

**Fig. 4:**
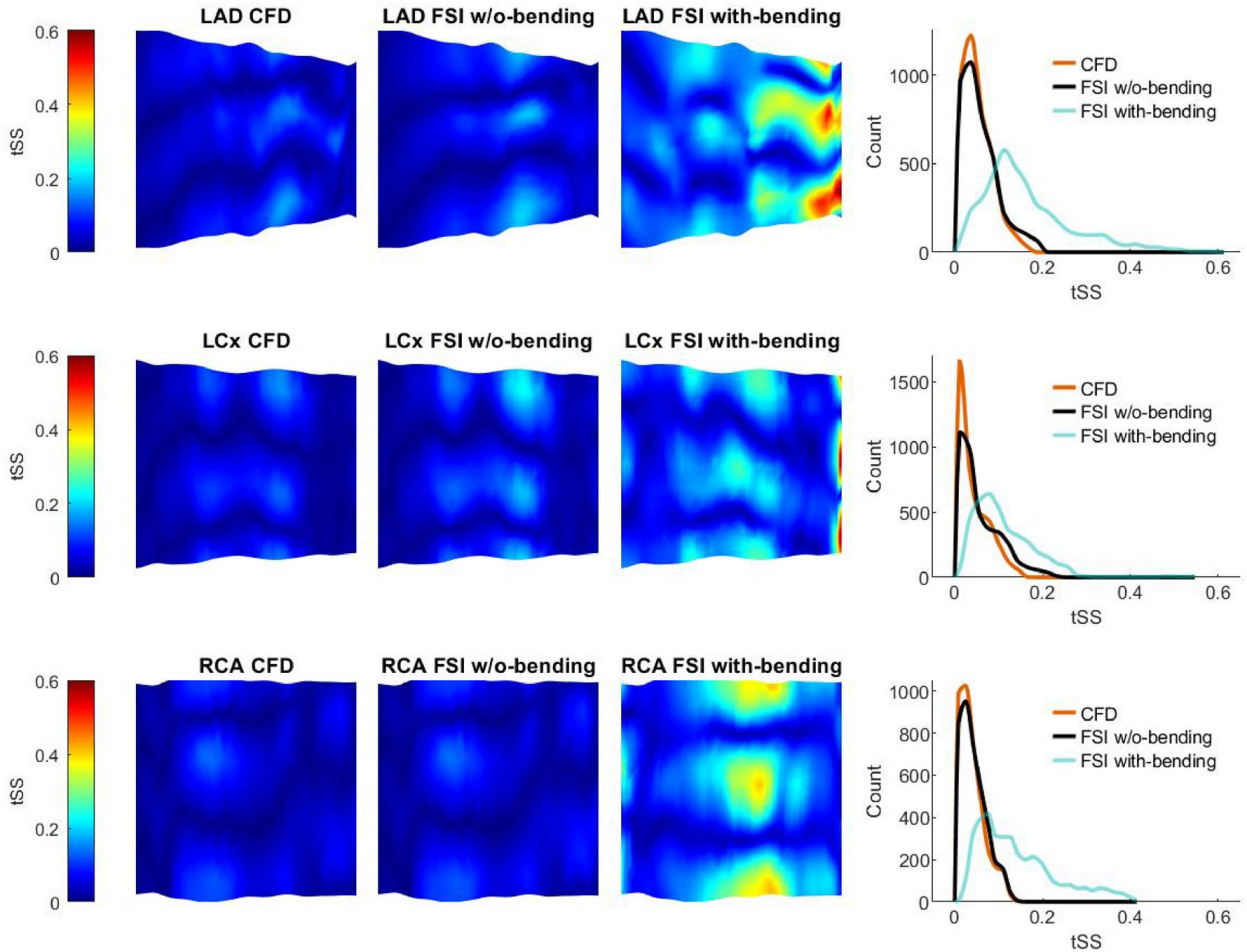
Results of transverse Shear Stress (tSS) for rigid-wall CFD, FSI without (w/o) bending, and FSI with-bending simulations, for LAD, LCx and RCA. Maps of each artery are presented as opened and flattened, showing magnitude and location of tSS for flow from proximal (left) to distal (right). Histograms compare the CFD, FSI without-bending, and FSI with-bending tSS datasets for each vessel

Implementation of FSI both without- and with-bending resulted in significant changes in all computed shear stress metrics compared to CFD (*p* = 0.0001). Parametric maps show differences in both magnitude and distribution of TAWSS between the FSI without-bending and CFD models for all three arteries (Fig. 3). Median values of TAWSS from FSI without-bending simulations are lower than from CFD, with the greatest difference in the LAD (− 5.7%) compared to the LCx (− 0.68%) and RCA (− 0.67%) (Table 1). Maximum TAWSS was reduced by 16.7% in the LAD but increased by 11.6% and 1.5% in the LCx and RCA, respectively. Changes in minimum TAWSS were more modest; + 1.5%, − 2.0% and − 4.9% for the LAD, LCx and RCA respectively. In all arteries, tSS values from the FSI without-bending models were higher than from CFD (Table 1, Fig. 4), increasing by 25%, 33% and 33% for the LAD, LCx and RCA respectively. OSI values were different in the FSI without-bending compared to the CFD models with the numerically greatest difference being observed in the LCx (Table 2). Maps showing the spatial distribution of OSI are shown in Supplementary Fig. 6.

**Table 1:** Shear stress metrics computed for rigid-wall CFD, FSI without-bending, and FSI with-bending simulations, for LAD, LCx and RCA

	LAD					LCx					RCA				
	Med	Q1	Q3	Min	Max	Med	Q1	Q3	Min	Max	Med	Q1	Q3	Min	Max
*TAWSS*															
CFD	1.40	1.12	2.16	0.66	4.18	1.48	1.29	1.66	1.02	2.41	1.49	1.28	1.69	0.81	2.63
Without-bending	1.32*	1.08	2.04	0.67	3.48	1.47	1.28	1.68	1.00	2.69	1.48	1.27	1.69	0.77	2.67
With-bending	1.45^$^*	1.17	2.38	0.48	4.19	1.60^$^*	1.40	1.87	0.65	3.38	1.45^$^*	1.23	1.65	0.73	2.37
*tSS*															
CFD	0.04	0.02	0.07	0.00	0.17	0.03	0.01	0.06	0.00	0.16	0.03	0.02	0.05	0.00	0.14
Without-bending	0.05*	0.02	0.08	0.00	0.20	0.04*	0.02	0.09	0.00	0.23	0.04*	0.02	0.06	0.00	0.14
With-bending	0.14^$^*	0.10	0.21	0.01	0.60	0.10^$^*	0.06	0.15	0.01	0.56	0.12^$^*	0.07	0.19	0.01	0.42

Metrics presented for Time Averaged Wall Shear Stress (TAWSS) and transverse Shear Stress (tSS)

*Med* median

*p < 0.0001 v CFD, ^$^*p* < 0.0001 v FSI without-bending

**Table 2:** Oscillatory Shear Index (OSI) computed for rigid-wall CFD, FSI without-bending, and FSI with-bending simulations, for LAD, LCx and RCA

	OSI				
	Median	Q1	Q3	Min	Max
*LAD*					
CFD	0.0003	0.0000	0.0008	0.0000	0.0025
Without-bending	0.0025*	0.0004	0.0088	0.0000	0.0348
With-bending	0.0077^$^*	0.0047	0.0117	0.0013	0.0336
*LCx*					
CFD	0.0001	0.0000	0.0006	0.0000	0.0039
Without-bending	0.0127*	0.0013	0.0417	0.0000	0.0835
With-bending	0.0127^$^*	0.0039	0.0274	0.0001	0.2255
*RCA*					
CFD	0.0002	0.0000	0.0005	0.0000	0.0038
Without-bending	0.0002*	0.0000	0.0008	0.0000	0.0042
With-bending	0.0054^$^*	0.003	0.0082	0.0002	0.0208

*p < 0.0001 v CFD, ^$^*p* < 0.0001 v FSI without-bending

### Computed Wall Shear Stress Metrics: FSI Without-Bending Compared to FSI With-Bending

Introduction of bending into the FSI simulation led to further changes in the spatial distributions (Figs. 3 and 4, Supplementary Fig. 6) and statistically significant differences in the magnitudes of computed shear stress metrics in all arteries (Tables 1, 2). Histogram plots of TAWSS distributions (Fig. 3) indicate the greatest differences were observed in the LAD and LCx. Median TAWSS values increased by 9.8% and 8.8% for the LAD and LCx, respectively. Notably, a 2% reduction in the median value of time-averaged wall shear was observed for the RCA (Table 1). Minimum values of TAWSS reduced by 28%, 35% and 5%, and maximum TAWSS values changed by 20%, 25% and − 11%, for the LAD, LCx and RCA, respectively.

Incorporation of bending led to more striking changes in tSS distribution (Fig. 4). Across all three arteries, median tSS increased (Fig. 4) being 180%, 150% and 200% higher compared to the FSI no-bending model for the LAD, LCx and RCA respectively (Table 1). Interestingly, the largest change in tSS was observed in the RCA, which by contrast showed the smallest difference in TAWSS (Table 1). Peak tSS values were markedly increased in the FSI with-bending models (Table 1). OSI results for the FSI with-bending show statistically significant changes in the distributions in all vessels (Table 2, Supplementary Fig. 6). Median values of OSI remained the same for the LCx, but increased by 208% and 2600% for the LAD and RCA, respectively.

### FSI Modelling of Arterial Strain

**Fig. 5:**
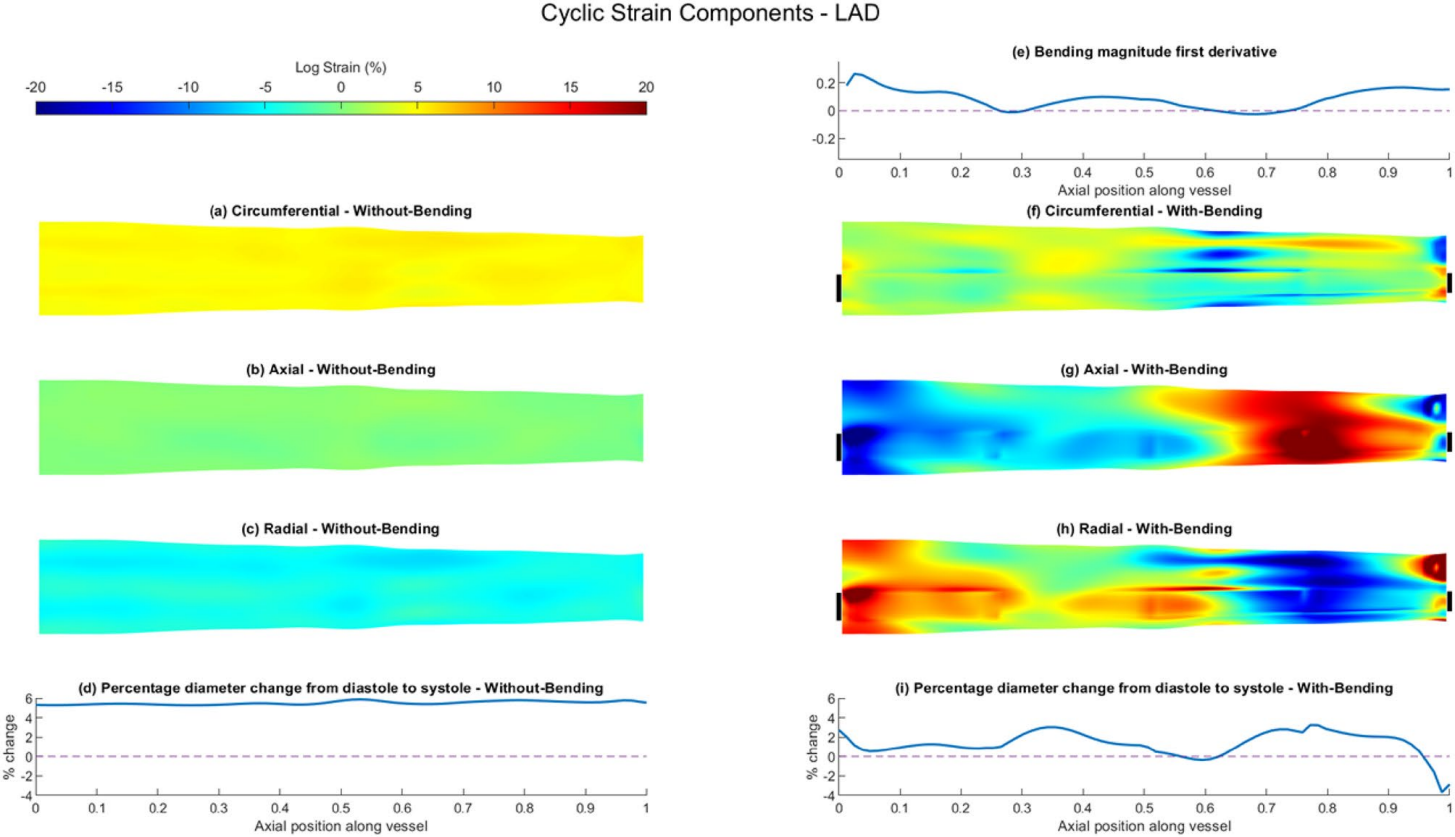
Summary of vessel wall response comparing results of FSI simulations of the LAD with- and without-bending simulations. Cyclic logarithmic strain (%) results for FSI without-bending simulation showing **a** circumferential, **b** axial and **c** radial components. **d** Percentage change of vessel wall diameter between diastole and systole along the length of the vessel for FSI without-bending simulation. **e** First spatial derivative of bending vector magnitude along the length of the vessel applied to FSI with-bending model. Cyclic logarithmic strain (%) results for FSI with-bending simulation showing **f** circumferential, **g** axial and **h** radial components. Circumferential location of bending loading application demarcated by black bars. **i** Percentage change of vessel wall diameter between diastole and systole along the length of the vessel for FSI with-bending simulation. All strain maps presented as opened and flattened, proximal region on the left, distal on the right

**Fig. 6:**
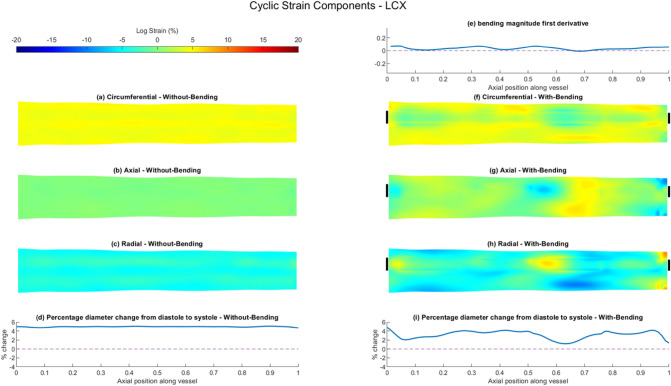
Summary of vessel wall response comparing results of FSI simulations of the LCx with- and without-bending simulations. Cyclic logarithmic strain (%) results for FSI without-bending simulation showing **a** circumferential, **b** axial and **c** radial components. **d** Percentage change of vessel wall diameter between diastole and systole along the length of the vessel for FSI without-bending simulation. **e** First spatial derivative of bending vector magnitude along the length of the vessel applied to FSI with-bending model. Cyclic logarithmic strain (%) results for FSI with-bending simulation showing **f** circumferential, **g** axial and **h** radial components. Circumferential location of bending loading application demarcated by black bars. **i** Percentage change of vessel wall diameter between diastole and systole along the length of the vessel for FSI with-bending simulation. All strain maps presented as opened and flattened, proximal region on the left, distal on the right

**Fig. 7:**
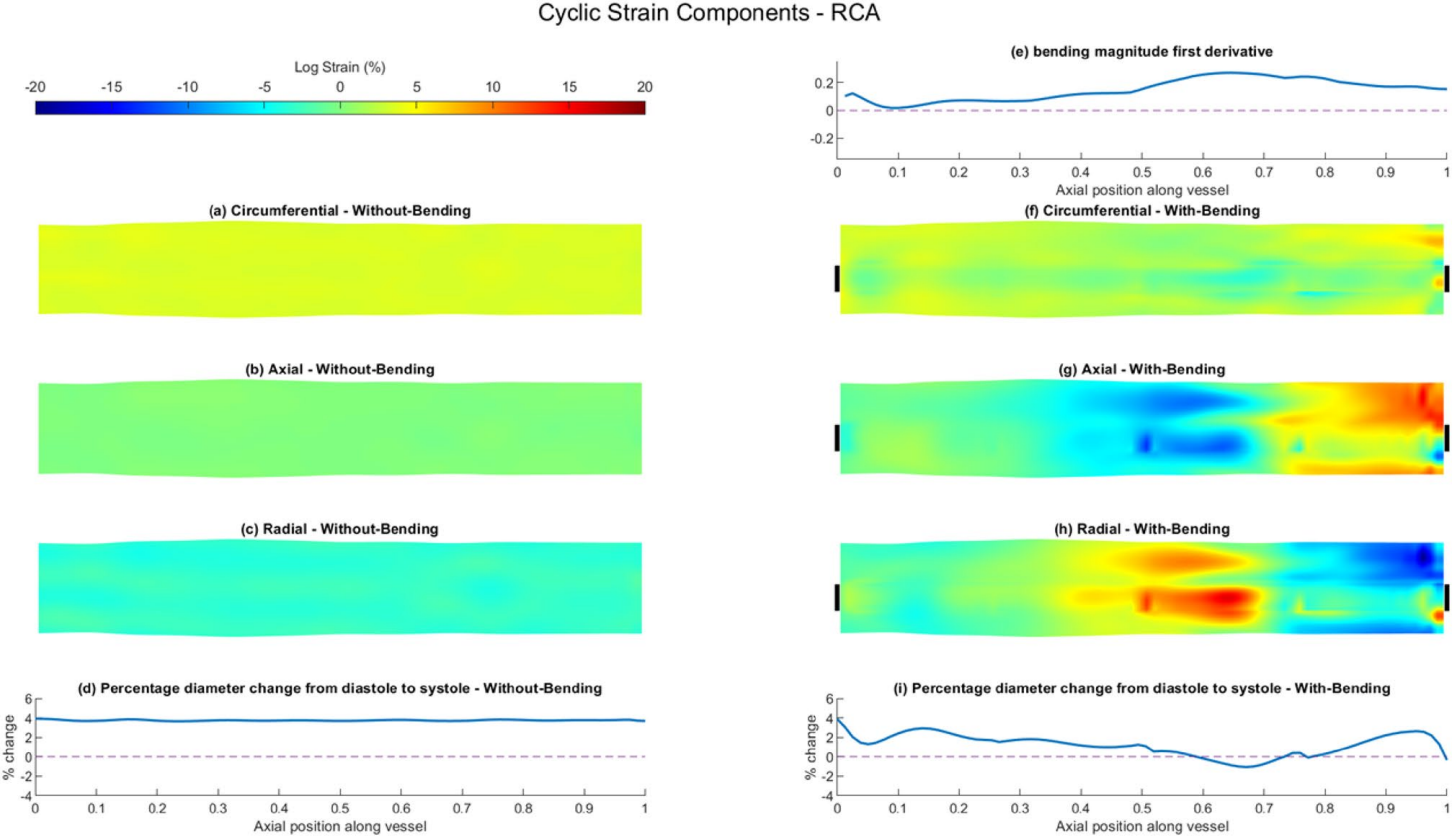
Summary of vessel wall response comparing results of FSI simulations of the RCA with- and without-bending simulations. Cyclic logarithmic strain (%) results for FSI without-bending simulation showing **a** circumferential, **b** axial and **c** radial components. **d** Percentage change of vessel wall diameter between diastole and systole along the length of the vessel for FSI without-bending simulation. **e** First spatial derivative of bending vector magnitude along the length of the vessel applied to FSI with-bending model. Cyclic logarithmic strain (%) results for FSI with-bending simulation showing **f** circumferential, **g** axial and **h** radial components. Circumferential location of bending loading application demarcated by black bars. **i** Percentage change of vessel wall diameter between diastole and systole along the length of the vessel for FSI with-bending simulation. All strain maps presented as opened and flattened, proximal region on the left, distal on the right

Results for the predicted artery-specific cyclic logarithmic strain and diameter change are shown in Figs. 5, 6 and 7. All three arteries show a similar modelled diameter change between systole and diastole in the FSI without-bending models, which varies little along the artery length, Figs. 5d, 6d and 7d. As all deformation in the no-bending models relates to arterial distension, the strain maps therefore show uniform, homogeneous strain fields in all three components, with negligible axial strain in all three arteries, (Figs. 5a–c, 6a–c and 7a–c, Supplementary Fig. 7). The latter is consistent with the applied boundary conditions for the FSI without-bending model. Median circumferential strains of 5.06% [4.90,5.23], 4.52% [4.36, 4.75], 3.51% [3.42, 3.61] for the LAD, LCx and RCA (Table 3) are consistent with the observed increases in arterial diameter at systole.

**Table 3:** Vessel wall cyclic logarithmic strain (%) computed for FSI without-bending and FSI with-bending simulations, for LAD, LCx and RCA, separated by component

	Without-bending (%)					With-bending (%)				
	Median	Q1	Q3	Min	Max	Median	Q1	Q3	Min	Max
*Axial strain*										
LAD	0.12	− 0.17	0.36	− 1.18	0.99	− 2.87	− 7.29	9.48	− 33.93	24.66
LCx	0.02	− 0.10	0.13	− 0.92	0.52	1.07*	− 0.36	2.24	− 9.77	5.98
RCA	0.02	− 0.09	0.13	− 0.59	0.43	− 1.13*	− 4.71	2.19	− 13.29	15.79
*Circumferential strain*										
LAD	5.06	4.90	5.23	4.14	5.82	1.12*	− 1.08	3.01	− 23.89	12.97
LCx	4.52	4.36	4.75	3.78	5.28	3.59*	2.16	4.37	− 4.52	5.94
RCA	3.51	3.42	3.61	3.10	4.11	1.80*	0.75	2.66	− 5.09	7.85
*Radial strain*										
LAD	− 4.46	− 5.00	− 4.09	− 6.48	− 2.75	1.35*	− 6.56	7.17	− 21.41	31.70
LCx	− 4.08	− 4.54	− 3.60	− 5.41	− 1.63	− 3.92*	− 5.30	− 2.08	− 10.24	9.24
RCA	− 3.07	− 3.35	− 2.80	− 4.26	− 1.81	− 0.40*	− 2.94	3.40	− 19.57	16.06

*p < 0.0001 v FSI without-bending

By contrast, the introduction of bending results in strongly heterogeneous strain fields for all three strain components and the strain maps show noticeable increases in the maximum strain values, particularly for the LAD and RCA (Figs. 5f–h, 6f–h and 7f–h, Supplementary Fig. 7). Axial cyclic strain is now apparent in all vessels, with median values of − 2.87% [− 7.29, 9.48], 1.07% [− 0.36, 2.24] and − 1.13% [− 4.71, 2.19] for the LAD, LCx and RCA respectively, suggesting that the LAD experiences a greater range of axial strain, and the LAD and RCA undergo more compressive strain (Table 3). Axial and radial strain components are seen to be interrelated in the strain maps, with regions of high tensile strain in the axial direction corresponding to high compressive strains in the radial direction, and vice-versa, Figs. 5g, h, 6g, h and 7g, h. Median values of radial strain remain similar to the without-bending case for the LCx, changing by only 3.92%, compared to changes of 130% for the LAD and 87% for the RCA. The strain fields in all directions under bending are seen to differ in both magnitude and distribution between each of the three arteries, which reflects the artery-specific application of bending loading.

Comparing axial variation in the bending magnitude derivatives for the three arteries (Figs. 5e, 6 e and 7e) highlights the difference in the nature of the bending loading, with each artery experiencing a differing proportion of bending deformation and rigid-body translation (Supplementary Fig. 4). This can also be observed in the systolic and diastolic configurations shown in Fig. 1. The FSI with-bending strain maps suggest lower minimum and maximum strain values in the LCx compared to the LAD and RCA, consistent with the smaller inter-quartile ranges in the axial and radial directions (Table 3). This is due to the lower proportion of bending vs translation in the applied bending loading, indicated by lower bending derivative values. Conversely, the LAD has the highest minimum and maximum strain values in all three components, with values of − 33.93% and 24.66% in the axial direction being the highest. Areas of high/low strain in the axial and radial directions in the LAD correspond approximately to the regions between the two local minima in the bending derivative (Fig. 5). For the RCA, strain minima and maxima in the axial and radial components lie within the distal half of the artery, where higher bending derivative values are seen (Fig. 7).

Unlike the FSI without-bending model, arterial dilatation is no longer uniform along the arterial length; introducing bending is seen to noticeably reduce dilatation, Figs. 5i, 6i and 7i. In the LAD and RCA, cyclic motion results in compression of the artery in some locations, leading to a decrease in diameter at systole compared to diastole. The largest diameter reduction, ~  − 4%, is seen in the most distal LAD (Fig. 5i), whereas the maximum diameter increment is 3.25%. The spatial distribution of the circumferential strain maps closely follows the trend in distension, Figs. 5f, 6f and 7f.

## Discussion

This study presents a FSI modelling framework for artery-specific modelling of in vivo biomechanics using experimentally acquired clinically-available source data. The major findings of this study are: (i) a vessel-specific coronary artery FSI model was created and verified against experimental data; (ii) the implementation of FSI leads to significant changes in the values and distribution of biologically relevant wall shear metrics compared with CFD; (iii) implementation of coronary artery motion results in significant changes in the spatial distribution and magnitude of shear stress metrics and cyclical strain.

### Verification

FSI simulation without-bending of the LAD using directly-measured haemodynamic boundary conditions was seen to provide physiologically relevant results for the flow behaviour and artery wall response, including artery wall distension of 5.3% consistent with experimental IVUS measurements of 4.5% ± 1%. Whilst in-vivo verification measurements were made, they were taken only at the proximal part of the LAD and not at multiple axial locations in the coronary artery. They could not, therefore, be used to verify the observed variation in vessel diameter predicted by the FSI with-bending models. To our knowledge, few FSI studies of healthy coronary vessels based on intravascular imaging modalities are reported in the literature with none showing direct experimental verification of vessel wall distension.

### FSI Without-Bending vs CFD

Vessel-specific FSI simulations without bending of three coronary arteries have been simulated and compared to equivalent CFD models, revealing changes in shear stress metrics which are important with respect to atherogenesis. Comparison of FSI without-bending results to rigid-wall CFD showed lower median values of TAWSS across all vessels, consistent with the literature and our previous work [7, 27]. Reductions in median TAWSS of 5.7% observed for the LAD are similar to those reported by Malve et al. [23]. Additionally, changes of − 16.7%/1.5%, 11.6%/ − 2%, 1.5%/ − 4.9% in maximum/minimum TAWSS were observed for the LAD, LCx and RCA respectively.

Our study also revealed changes in tSS and OSI (Tables 1 and 2), which have not been widely reported previously. Magnitudes of tSS and OSI for the CFD models were consistent with those observed for CFD simulations in mildly diseased porcine coronaries by Hoogendoorn et al. [14]. Changes in shear metrics relating to multidirectional flow are of note as these markers are considered to be of biological importance [1, 14, 24, 30].

### FSI With- and Without-Bending: Influence on Shear Metrics

We successfully included bending into the artery-specific simulation of porcine LAD, LCx and RCA coronary arteries, which led to artery-specific changes in wall shear stress metrics TAWSS, tSS and OSI, highlighting the need for artery specificity for such analyses.

Few comparable studies are available for FSI on the influence of coronary bending on healthy vessels in the literature, and to our knowledge changes in multidirectional flow have not been reported. Torii et al. [46] found increases of mean TAWSS and OSI when comparing static CFD simulations to rigid-wall CFD with bending for a human RCA, as well as changes in the spatial distribution of TAWSS. Hasan et al. [11] report no influence of bending on shear stress parameters in their FSI study of an idealised LAD geometry. For stenotic vessels, Yang et al. [51] reported a 15% decrease in flow maximum shear stress from an FSI analysis of a stenotic vessel with bending. Ramaswamy et al. [36] found reduced TAWSS but increased OSI with bending in a CFD-only study of a stenotic LAD. Given the importance of causal associations between shear metrics in atherogenesis, particularly TAWSS, tSS and OSI, the results support future evaluation of an FSI-based modelling approach which includes bending.

### FSI With- and Without-Bending: Effect on Vessel Wall Strain

The use of FSI simulations instead of rigid-wall CFD enables the mechanical behaviour of the vessel wall to be considered in addition to the fluid shear stress. Cyclic strain has been suggested to influence EC phenotype and function via a number of pathways including endothelial to mesenchymal transition which is a mechanism that has been implicated in a number of cardiovascular diseases, including atherosclerosis [15]. The FSI without-bending models presented cyclic strain fields in all three directions which were homogenous, and vessel dilation was consistent along the axial length of all three arteries. Strain levels in the radial and circumferential direction were consistent with the dilation of the vessel, and strain in the axial direction was negligible. The introduction of bending causes marked changes to the strain magnitudes and distribution in all three vessels. Inclusion of bending provides an additional mechanical loading to that of the blood pressure, altering all components of the strain fields to become highly heterogenous. Axial strain was no longer minimal with bending applied, showing areas of both tension and compression in all vessels. For example, the maximum and minimum axial strain values changed by up to + 24.7% and − 33.9% for the LAD (Table 3). Median values for circumferential and radial directions were reduced in the with-bending models for all vessels, but minimum and maximum values show a much wider range of strains. This is particularly true for the LAD and RCA, which the first derivatives of the bending displacements show to undergo a greater proportion of bending deformation than the LCx.

Differences in response between vessels after inclusion of bending are noted for both shear and strain metrics. Variation in bending displacements calculated in this study are consistent with previous experimental observations. Konta and Bett [19] categorised coronary bending into three types of motion: ‘Bend’, ‘Lever’ and ‘Parallel D’, with an individual vessel’s motion having a different combination of those three components. Javadzadegan et al. [16] showed a difference in WSS and the presence of disturbed flow by varying the type of motion applied to an FSI simulation of an LAD using Konta and Bett’s classifications. Ding and Friedman [4, 5] analysed biplane coronary angiograms to show that the total displacement of the RCA is approximately twice that of the LAD, but the torsion and twisting is greater in the LAD. They also observed that the motion parameters vary not just between vessels but also along the length of the vessel. Similarly, Kataria et al. [18] using enhanced CT showed that the LCx has greater displacement than the LAD, and the RCA a larger displacement than the LCx. These findings are consistent with our results, with heterogenous strain and shear maps demonstrating axial variation in those properties, and localised strain concentrations observed in areas of higher bending. As expected, the difference between vessels in proportion of bending and translation, and axial variation in bending loading, is reflected in the results. The results therefore suggest the inclusion of coronary motion, including bending, provides a more physiologically accurate representation of the mechanical loading experienced by the vessel wall. Results also highlight the importance of vessel-specificity to study the correct configuration of vessel geometry, bending and flow properties.

The application of a coronary bending loading on an artery model inherently presents a challenge, as without modelling the heart muscle upon which the vessels lie, an artificial loading is required. Within the FSI models presented here, coronary bending is applied as a nodal displacement on the outer surface of the vessel, which prevents distension of that part of the vessel wall under haemodynamic pressure loading, potentially having a local effect on the shear stress and wall strain. The region of nodal loading is restricted to ~ 30% of the circumference to limit the effect on vessel expansion, and to mimic the siting of the vessel on the surface of the heart. This is valid for the proximal portion of the arteries studied here, but a value closer to ~ 50% may be more appropriate for the mid and distal regions.

A second limitation relates to the variation of predicted distension over the vessel length under cyclic bending. In some circumstances, a localised reduction, rather than increase in arterial diameter is observed. It is not clear if this is a physiological or methodological effect. IVUS measurements acquired during this study were recorded in the proximal part of one vessel only, and limited information is available on the variation in coronary artery diameter over the course of the cardiac cycle. A methodological explanation may be the difficulty in accounting for any change in vessel length when co-registering the angiogram centrelines at diastole and systole. Correcting for this may enable more accurate bending displacements to be calculated. Additionally, coronary bending displacements were calculated from centreline reconstructions of the un-instrumented vessel, which were then applied to a model reconstructed from an instrumented artery. Therefore, any discrepancy between the instrumented and un-instrumented vessel geometries could contribute to errors in the bending loading. This may warrant further investigation. It is also noted that bending was based on changes in the vessel centreline, so no torsion loading was applied to the vessel, due to the difficulty in tracking the detailed 3D position of the artery walls over time.

Whilst pre-stress relating to the inherent axial stretch and baseline blood pressure have been incorporated into the model, the residual stress due to the opening angle has not, due to the difficulty in simulating the large displacements associated with the typical opening angle of porcine vessels. However, these stresses are likely to be small compared to those arising from axial pre-stress and blood pressure [43].

Finally, the exclusion of side-branches and bifurcations from the vessel geometries is a simplification, which may result in less disturbed flow both with- and without-bending. The magnitude of the OSI and tSS may therefore be underestimated. It is noted, however, that significant changes are observed in the RCA, which typically has far fewer bifurcations than the LAD and LCx.

In conclusion, this study has presented a methodology for subject- and vessel-specific FSI modelling of coronary arteries, utilising directly measured boundary conditions. The results show the differences in shear metrics between CFD and FSI models. The incorporation of coronary bending has been successfully applied, to provide a more physiological representation of the biomechanics of coronary arteries, which reveals changes in biologically significant wall shear metrics and cyclic strain. Importantly, these changes are shown to vary independently across the LAD, LCx and RCA, outlining the difference in the physiological environment of each artery. This reinforces the importance of vessel-specific coronary artery biomechanical modelling, to ensure that the correct physiological loadings are applied, and the biomechanics correctly assessed. This may be particularly important when considering the relationship between local arterial biomechanics and atherogenesis in experimental and clinical studies. We provide a framework for clinical translation of vessel-specific FSI modelling, which has potential to provide new insights into the biomechanical basis for atherogenesis.

## Supplementary Information

Below is the link to the electronic supplementary material.

Supplementary file1 (DOCX 4093 KB)
