# Supplementary material for: Considering the Influence of Coronary Motion on Artery-Specific Biomechanics Using Fluid–Structure Interaction Simulation
Source: Ann Biomed Eng. 2023 Jul 12;51(9):1950–64. doi: 10.1007/s10439-023-03214-0 (PMC10409843; doi:10.1007/s10439-023-03214-0)
Supplement: Supplementary file 1 — Supplementary file1 (DOCX 4093 KB) [file 10439_2023_3214_MOESM1_ESM.docx]

# Supplementary material

## Methods

## Animal Experimental Procedures

All animal procedures were conducted in accordance with institutional guidelines of the Griffin Institute, London, UK and the Guide for the Care and Use of Laboratory Animals (Institute of Laboratory Animal Resources, 1996). All experimental procedures received the approval of the UK Home Office (PPL 70/7765).

Experimental studies were performed in transgenic *D374Y*-PCSK9 hyperlipidaemic minipigs (n=5) fed a cholate free high fat high cholesterol diet (Test Diet) as previously reported^2^. Before cardiac catheterisation procedures, animals were initially premedicated with oral diazepam (1mg/kg) and acepromazine (1mg/kg) after which intramuscular ketamine (5mg/kg) and xylazine (1mg/kg). After endotracheal intubation, animals were mechanically ventilated with isoflurane (1-3%) as an inhalational anaesthetic. Vital signs including end-tidal CO2, oxygen saturations, arterial blood pressure and electrocardiogram were monitored continuously. At each instrumentation, animals received 1g ampicillin intravenously. The day before stent implantation, animals received clopidogrel 600mg and aspirin 300mg. Following stent implantation, animals received daily aspirin 75mg and clopidogrel 150mg orally for the study duration.

Cardiac catheterisation was performed via an 8 French right femoral sheath (Arrow International) placed percutaneously under ultrasound guidance. Unfractionated heparin (5000u) was administered with a further 1000u administered every hour. The left and right coronary arteries were engaged with an 8F Hockey Stick I guiding catheter. Coronary angiography was performed using a single plane X-ray fluoroscopy system (Innova 4100, GE Healthcare). Angiograms were acquired after administration of intracoronary isosorbide dinitrate (0.1-0.3mg) in orthogonal views (>30^o^ separation) with respiration briefly held in end-expiration to minimise respiratory motion artefact using iodinated contrast (iodixanol 320 mgI/mL, GE Healthcare, UK) with views selected to minimise vessel overlap.

Inlet coronary blood velocity was measured in each coronary artery using a Combowire (Volcano Corporation, San Diego, CA, USA). Equalisation was performed at the guide catheter tip. Measurements were undertaken >5mm from a significant sidebranch (> 2mm diameter) for 10-20 cycles. Intracoronary frequency domain optical coherence tomography (OCT) was performed with an Illumien system and Dragonfly-2 catheters (Abbott Vascular). Before OCT image acquisition, the OCT catheter position was imaged in orthogonal views during injection of iodinated contrast to enable 3-D reconstruction of the catheter path within the coronary artery. OCT pullbacks were performed at 20mm/s during pump injection of iodixanol (4-8mL/s).

## Reconstruction of 3D coronary arterial geometries

OCT-based vessel reconstruction methodology was performed as previously reported ^1, 2, 4, 6, 8, 9^. Coronary lumen contours were segmented using CAAS Intravascular Software (PieMedical, Rotterdam, NL). Only clear image frames with visible lumen and vessel >270^o^ continuous arc were analysed. Whenever a side branch was present in the OCT image, the lumen was segmented as if no side branch was present.

A summary of the workflow used to prepare computational simulations of coronary arteries from vessel reconstructions based on the fusion of OCT and angiograms is as follows:

1. An OCT pullback of the vessel is manually contoured to provide coordinates of the outline of the lumen. A subset of the frames is selected corresponding to the vessel Region Of Interest (ROI), based upon anatomical landmarks.
2. The OCT catheter outline is identified in sequential orthogonal angiograms and Quantitative Coronary Angiography using CAAS 3D QCA software (PieMedical, Rotterdam, NL) is used to perform a 3D reconstruction of the catheter.
3. Stl geometry of catheter generated from QCA is used to produce catheter path coordinates using VMTK lab software
4. The catheter path is smoothed, and a 3D reconstruction of the vessel is computed from the contoured OCT output and catheter path coordinates. This is performed using in-house MATLAB code.
5. The reconstructed vessel lumen geometry is smoothed.
6. Reconstructed vessel lumen is used to generate the solid mesh and input file for the Abaqus/Standard implicit solid solver.
7. Reconstructed vessel lumen is used to generate the fluid mesh and input file for the Abaqus/CFD fluid solver.
8. Combowire raw measurement data of in-vivo blood pressure and flow velocity time-series is smoothed and synchronised using concurrent ECG data. Processed values are inserted into Abaqus/CFD input file for use as directly measured fluid boundary conditions.
9. For FSI models with cardiac bending loading, a 3D QCA reconstruction from sequential orthogonal angiograms is computed for the vessel at diastole and systole. Vessel centrelines from each state are calculated from the reconstructed vessel stl geometry. The difference between the two centrelines is calculated and used to generate the bending displacement loading, which is input into the solid Abaqus/Standard input file.
10. The FSI simulation is run on the High Performance Computing (HPC) facility at Imperial College London
11. Shear and strain metrics are generated from the simulation results.

### Mesh generation

Meshing of both the fluid and solid domains was performed using an in-house MATLAB script. Linear hexahedral elements were used throughout. All models were discretised in the same structured way, using 80 elements in the axial direction and 48 elements in the circumferential direction. The fluid domain mesh incorporated a 3 element boundary layer mesh of growth factor 1.2 (Supplementary Figure 1). The artery wall and perivascular material had 4 and 3 elements through thickness, respectively (Supplementary Figure 1). The mesh for the fluid and solid domains was continuous and nodes were shared at the fluid-structure interface. To facilitate the radial dilatation of the artery under loading from the blood flow within FSI simulations, automatic mesh movement was applied to the fluid mesh to enable it to expand with the movement of the artery wall. This movement is automatically calculated by the fluid solver using an Arbitrary Lagrange-Euler (ALE) approach.

The vessel wall (structural domain) is discretized using fully-integrated linear 8-noded hexahedral elements. Owing to the application of a nearly-incompressible hyperelastic constitutive model a hybrid element formulation, C3D8H, is used, which includes an additional independently-interpolated variable for pressure. For the perivascular support material, a hybrid element formulation is not required, and so standard fully-integrated linear 8-noded elements are used with formulation C3D8. For the fluid domain, linear 8-noded hexahedral elements with an ALE formulation, FC3D8, are used.

## Structural Boundary Conditions

The structural boundary conditions are detailed in Supplementary Table 1 for the three simulation types; rigid CFD, FSI without-bending and FSI with-bending. Example construction of the fluid and solid domains is provided in Supplementary Figure 1. The node sets on the inlet/outlet faces used for the constraints of the fluid and solid domains for the FSI models are shown in Supplementary Figure 2. For the without-bending case, single point constraints are applied to the nodes in the node set, as described in Supplementary Table 1. For the with-bending models, the node sets are used as a sliding contact surface with a rigid plane, to ensure all nodes on the face stay on the same plane whilst permitting the inlet/outlet faces to move with the bending displacement. This is achieved using the *Contact Pair and *Surface Interaction keywords within Abaqus/Standard^5^. Supplementary Figure 3 shows an example of the node set on the outer surface of the perivascular support material, used to constrain the FSI without-bending model, and to apply bending displacement for the FSI with-bending model, as per Supplementary Table 1.

**Supplementary Table 1**

| **Rigid CFD** | |
| --- | --- |
| Lumen walls, inlet and outlet faces. | Lumen geometry completely constrained in all directions (rigid wall). |
| **FSI without bending** | |
| Lumen and vessel wall | No constraints applied - free to dilate under pressure loading from fluid domain. |
| Perivascular material | Outer surface – nodal constraints in all directions. |
| Inlet face (fluid domain, vessel wall, perivascular material) | All nodes constrained in axial and circumferential direction – radial movement free. |
| Outlet face (fluid domain, vessel wall, perivascular material) | All nodes constrained in axial and circumferential direction – radial movement free. |
| **FSI with bending** | |
| Lumen and vessel wall | Prescribed bending motion applied over 30% of vessel circumference, to nodes on outer surface of vessel wall.  Remainder of nodes free to dilate under pressure loading from fluid domain. |
| Perivascular material | Prescribed bending motion applied to all nodes on outer surface of perivascular material. |
| Inlet face (fluid domain, vessel wall, perivascular material) | All nodes constrained to rigid-body plane via sliding contact, enforcing axial constraint but allowing radial movement. Bending displacement applied to rigid-body plane, ensuring that inlet face translates under bending loading. |
| Outlet face (fluid domain, vessel wall, perivascular material) | All nodes constrained to rigid-body plane via sliding contact, enforcing axial constraint and allowing radial movement. Bending displacement applied to rigid-body plane, ensuring that outlet face translates under bending loading. |

**Supplementary Table 1**: Summary of structural constraints applied for rigid CFD, FSI without-bending and FSI with-bending simulations

Observed bending displacements vary according to axial position within the vessel, and hence are applied as 3D nodal displacements specific to the axial location of the node, in a row by row basis. An example of the nodal locations for the bending displacements is shown in Supplementary Figure 5.

## Reproduction of results

Input files have been made available for the three different analysis types of the Left Anterior Descending artery: rigid-wall Computational Fluid Dynamics (CFD), Fluid Structure Interaction (FSI) without-bending, and FSI with-bending.  The models are suitable for running in Abaqus/Standard and Abaqus/CFD v6.14. The files can be found here: https://doi.org/10.5281/zenodo.7654957.

## Calculation of shear metrics

Shear metrics were calculated according to the following equations.

Time Averaged Wall Shear Stress (TAWSS) ^7^:

$TAWSS= \frac{1}{T}\int_{0}^{T} \left| \vec{\tau_{\omega}} \right|dt$ A1

Where *T* represents the cardiac cycle time period, $\vec{\tau_{\omega}}$ the instantaneous WSS vector, and *t* the time.

Oscillatory Shear Index (OSI) ^7^:

$TAWSS= 0.5\left( 1-\frac{\left| \int_{0}^{T} \vec{\tau_{\omega}}dt \right|}{\int_{0}^{T} \left| \vec{\tau_{\omega}} \right|dt} \right)$ A2

Transverse Shear Stress (tSS) ^3^:

$\mathrm{tSS} = \frac{1}{T}\int_{0}^{T} \left| \vec{\tau_{\omega}} \cdot\left( \vec{n} \times\frac{\int_{0}^{T} \vec{\tau_{\omega}}dt}{\left| \int_{0}^{T} \vec{\tau_{\omega}}dt \right|} \right) \right|\mathrm{dt}$ A3

Where $\vec{n}$ represents the normal to the vessel wall surface.

## Calculation of computed vessel diameters

Given that the reconstructed vessel geometries will have non-circular cross-sections, calculated values of diameter are based on mean values. Diameters are calculated for each pair of opposite nodes on a circumferential section, which provides 24 values based on a circumferential mesh resolution of 48 nodes. The mean of these 24 diameter measurements is then calculated.

## References

1. Krams R., J. J. Wentzel, J. A. F. Oomen, R. Vinke, J. C. H. Schuurbiers, P. J. d. Feyter, P. W. Serruys and C. J. Slager. Evaluation of Endothelial Shear Stress and 3D Geometry as Factors Determining the Development of Atherosclerosis and Remodeling in Human Coronary Arteries in Vivo. *Arteriosclerosis, Thrombosis, and Vascular Biology* 17: 2061-2065, 1997.

2. Pedrigi R. M., C. B. Poulsen, V. V. Mehta, N. Ramsing Holm, N. Pareek, A. L. Post, I. D. Kilic, W. A. Banya, G. Dall'Ara, A. Mattesini, M. M. Bjorklund, N. P. Andersen, A. K. Grondal, E. Petretto, N. Foin, J. E. Davies, C. Di Mario, J. Fog Bentzon, H. Erik Botker, E. Falk, R. Krams and R. de Silva. Inducing Persistent Flow Disturbances Accelerates Atherogenesis and Promotes Thin Cap Fibroatheroma Development in D374Y-PCSK9 Hypercholesterolemic Minipigs. *Circulation* 132: 1003-1012, 2015.

3. Peiffer V., S. J. Sherwin and P. D. Weinberg. Computation in the rabbit aorta of a new metric - the transverse wall shear stress - to quantify the multidirectional character of disturbed blood flow. *J Biomech* 46: 2651-2658, 2013.

4. Post A. L., P. Cernohorsky, R. M. Pedrigi, G. J. Streekstra, J. N. S. D'Hooghe, J. T. Annema, S. D. Strackee, R. Krams, T. G. van Leeuwen, D. M. de Bruin and D. J. Faber. 3D co-registration algorithm for catheter-based optical coherence tomography. *Osa Continuum* 3: 2707-2721, 2020.

5. Simulia D. S. Abaqus Analysis User's Guide, Version 6.14. 2014.

6. Slager C. J., J. J. Wentzel, J. C. Schuurbiers, J. A. Oomen, J. Kloet, R. Krams, C. von Birgelen, W. J. van der Giessen, P. W. Serruys and P. J. de Feyter. True 3-dimensional reconstruction of coronary arteries in patients by fusion of angiography and IVUS (ANGUS) and its quantitative validation. *Circulation* 102: 511-516, 2000.

7. Torii R., J. Keegan, N. B. Wood, A. W. Dowsey, A. D. Hughes, G. Z. Yang, D. N. Firmin, S. A. McG Thom and X. Y. Xu. The effect of dynamic vessel motion on haemodynamic parameters in the right coronary artery: a combined MR and CFD study. *Br J Radiol* 82 Spec No 1: S24-32, 2009.

8. Wentzel J. J., R. Krams, J. C. H. Schuurbiers, J. A. Oomen, J. Kloet, W. J. v. d. Giessen, P. W. Serruys and C. J. Slager. Relationship Between Neointimal Thickness and Shear Stress After Wallstent Implantation in Human Coronary Arteries. *Circulation* 103: 1740-1745, 2001.

9. Wentzel J. J., D. M. Whelan, W. J. van der Giessen, H. M. M. van Beusekom, I. Andhyiswara, P. W. Serruys, C. J. Slager and R. Krams. Coronary stent implantation changes 3-D vessel geometry and 3-D shear stress distribution. *Journal of Biomechanics* 33: 1287-1295, 2000.

## Supplementary Figures

**LAD**


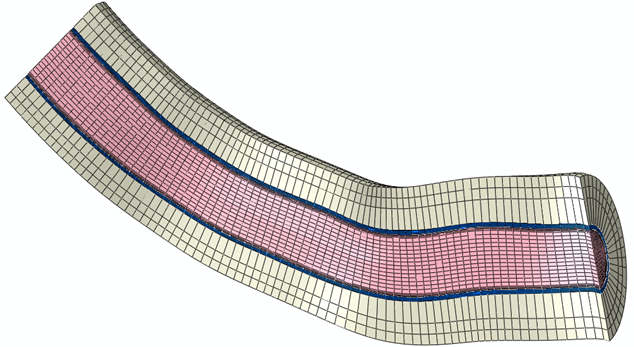


**LCx**


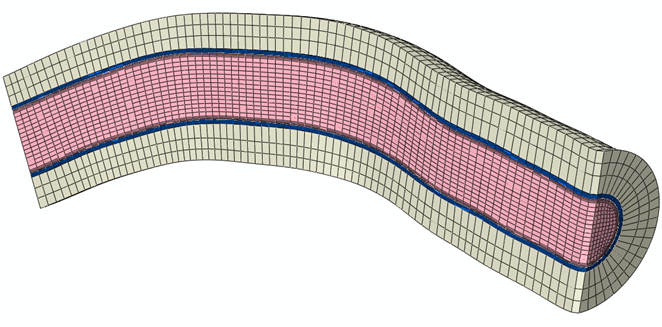


**RCA**


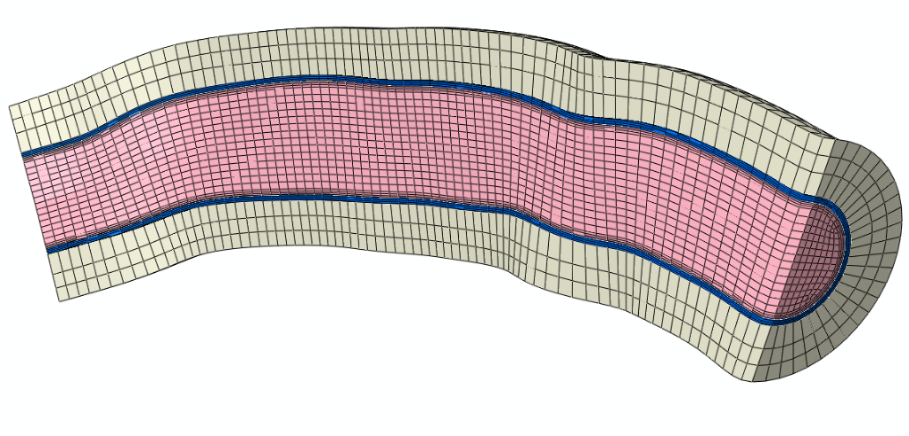


**Supplementary Figure 1**: Axial cross-sections showing combined fluid and structural domain FSI computational meshes for LAD, LCx, and RCA. Fluid domain shown in pink, vessel wall shown in blue, perivascular support material shown in off-white. Flow direction is from left to right, inlet extension not shown.


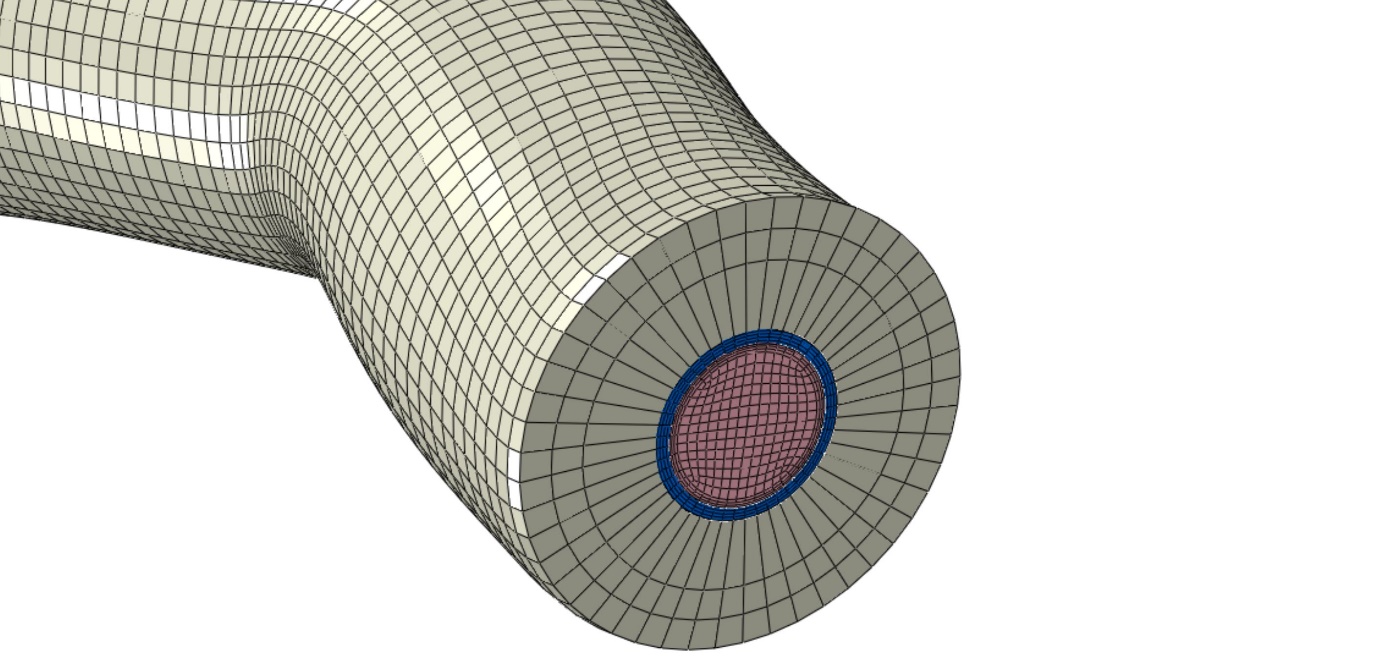

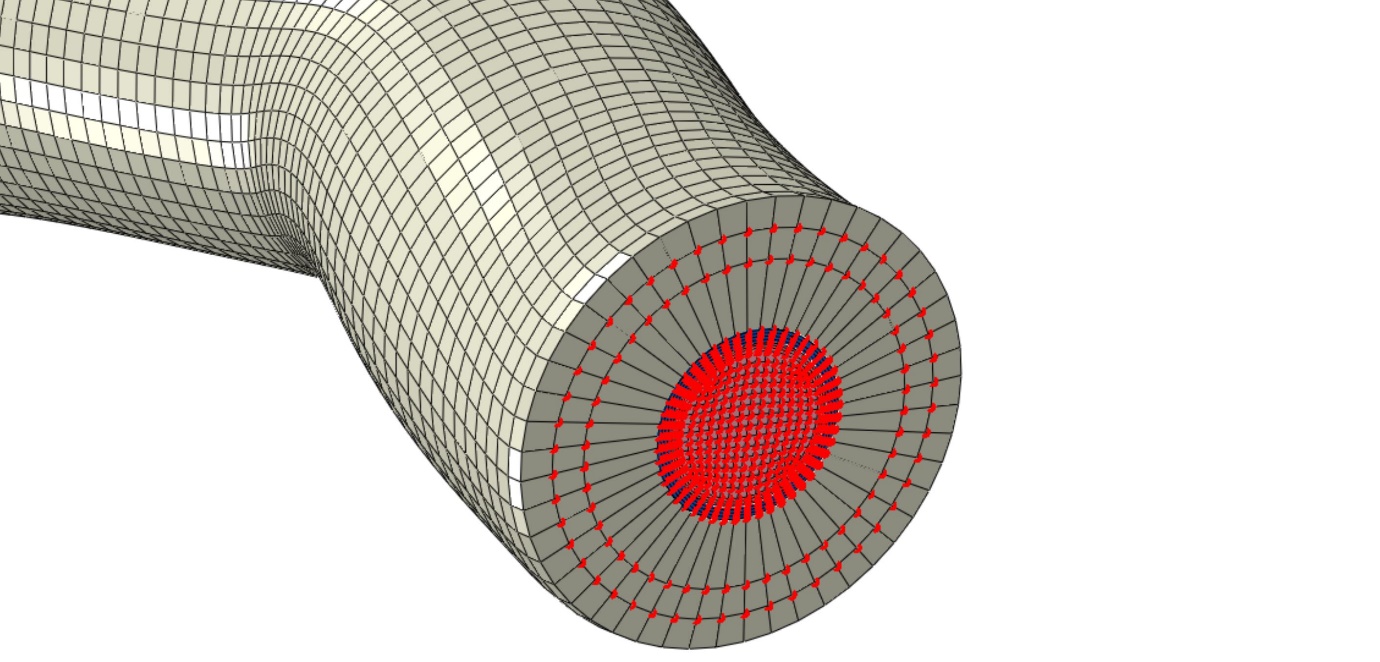


**Supplementary Figure 2**: Combined fluid and structural domain FSI computational meshes for LAD, showing outlet face without and with node set (marked in red) used for structural constraints. Node set on inlet face is identical. Fluid domain shown in pink, vessel wall shown in blue, perivascular support material shown in off-white.


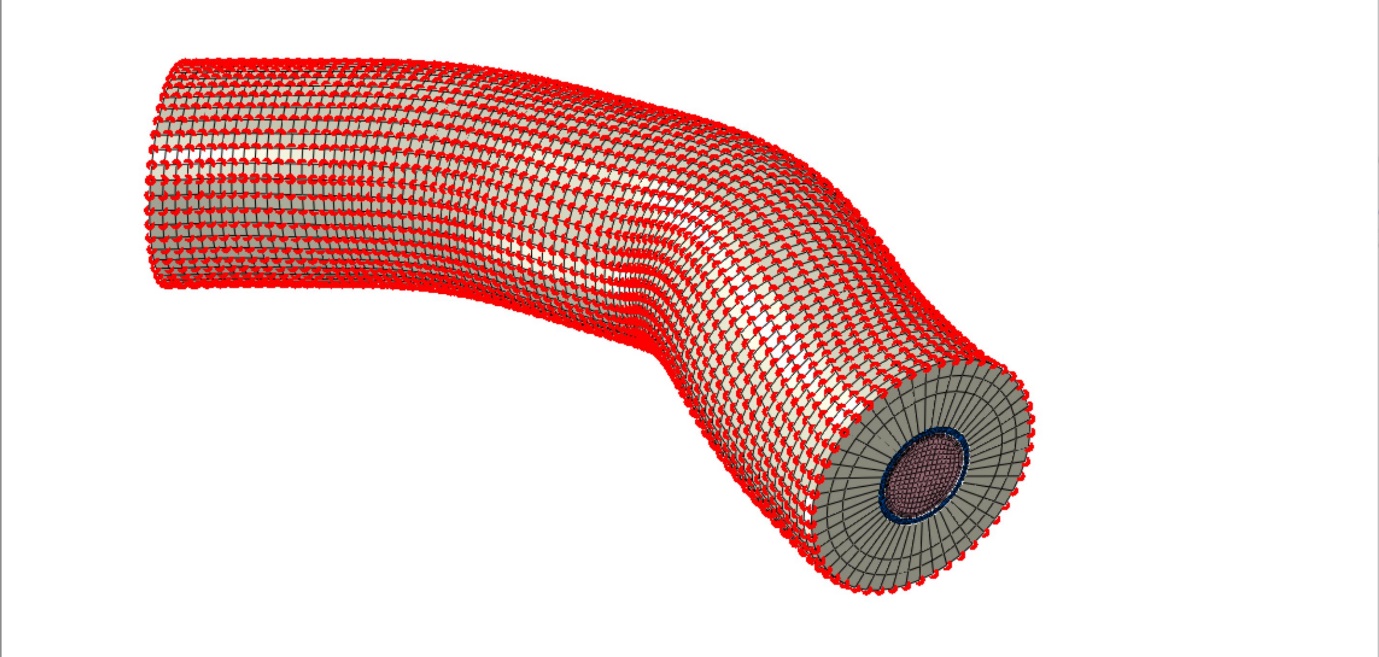


**Supplementary Figure 3**: Combined fluid and structural domain FSI computational meshes for LAD, showing node set, marked in red, used for structural constraints on outer surface of perivascular support material. Fluid domain shown in pink, vessel wall shown in blue, perivascular support material shown in off-white.


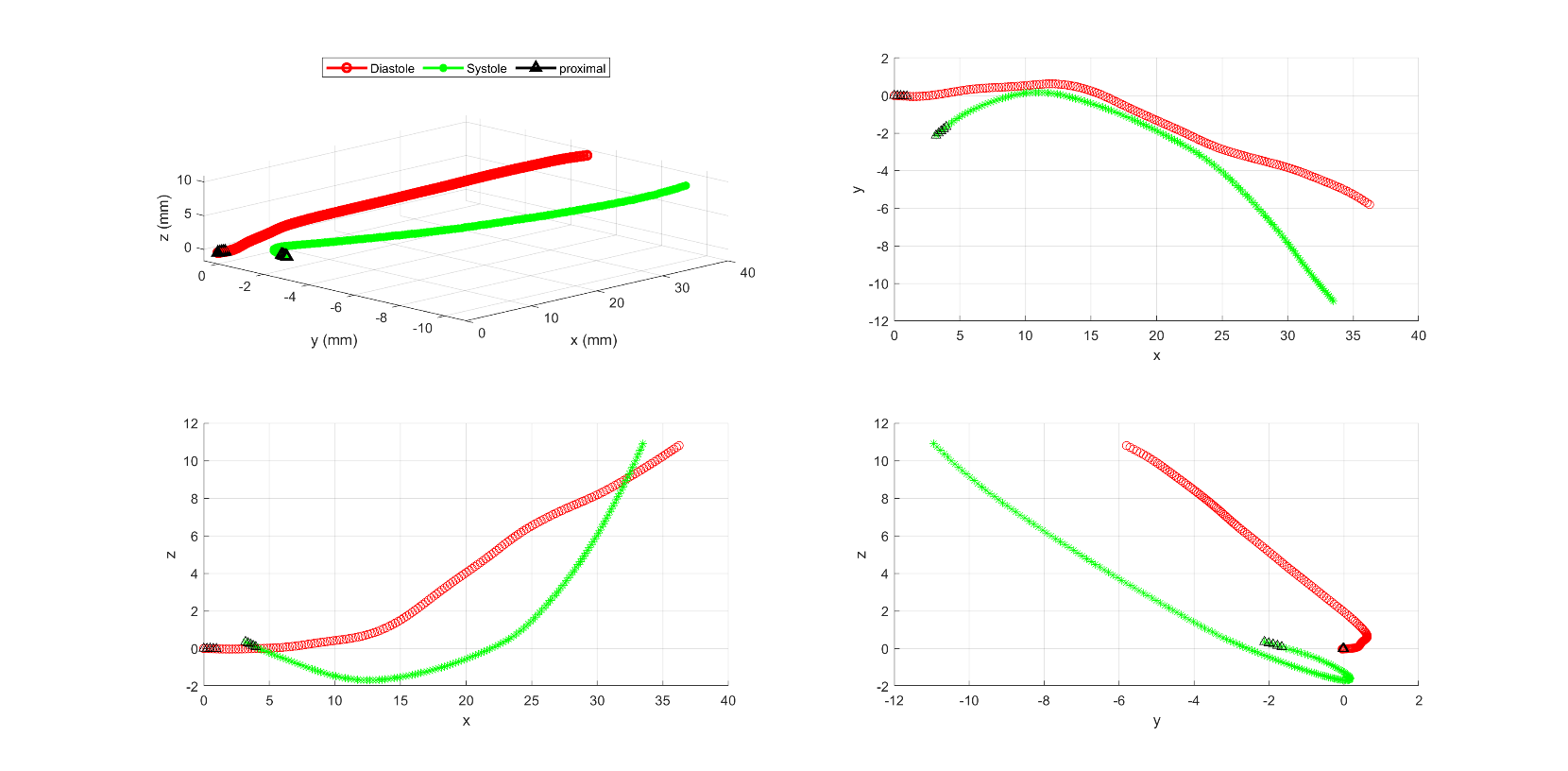


**LAD**

**LCx**


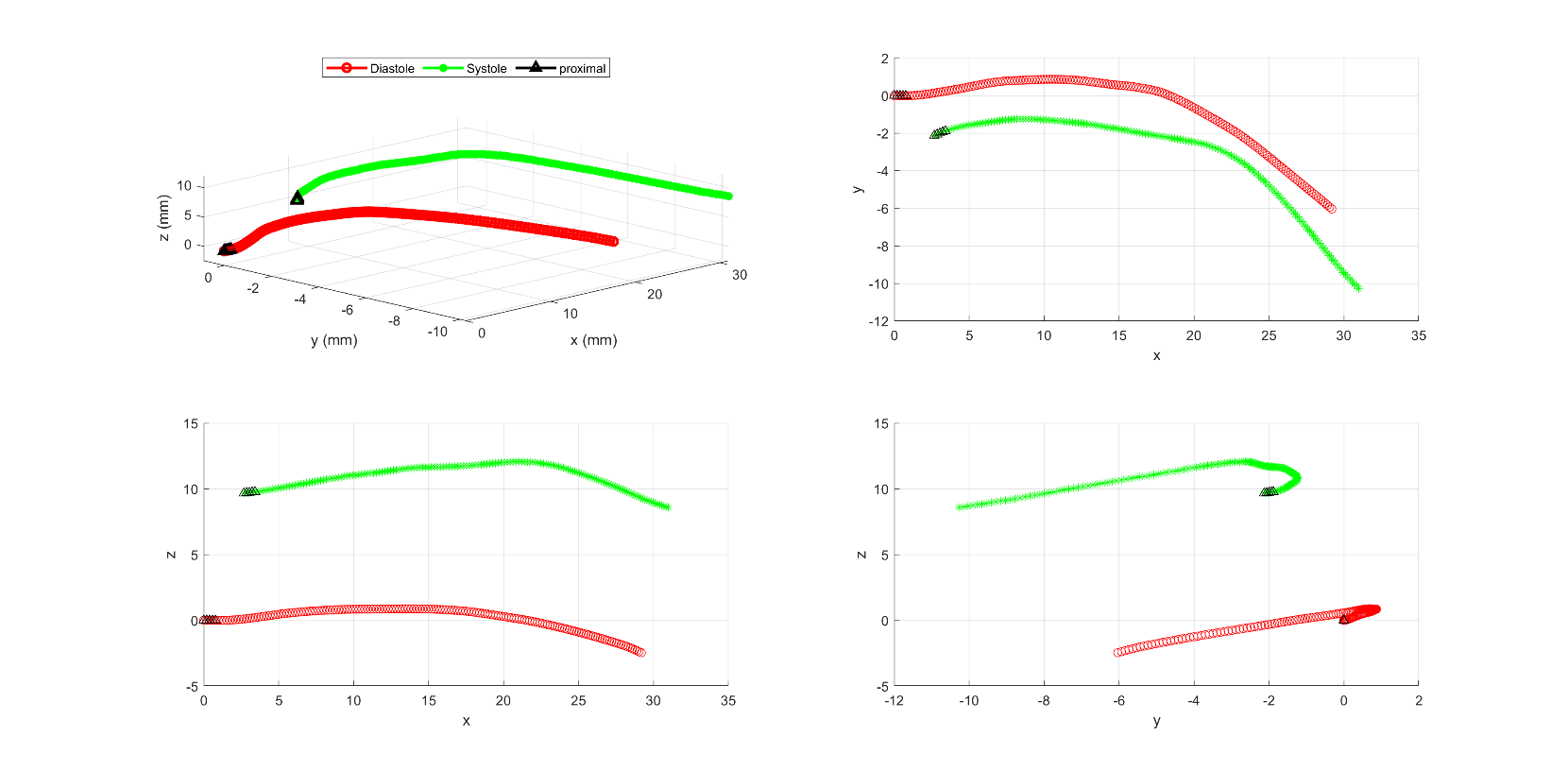


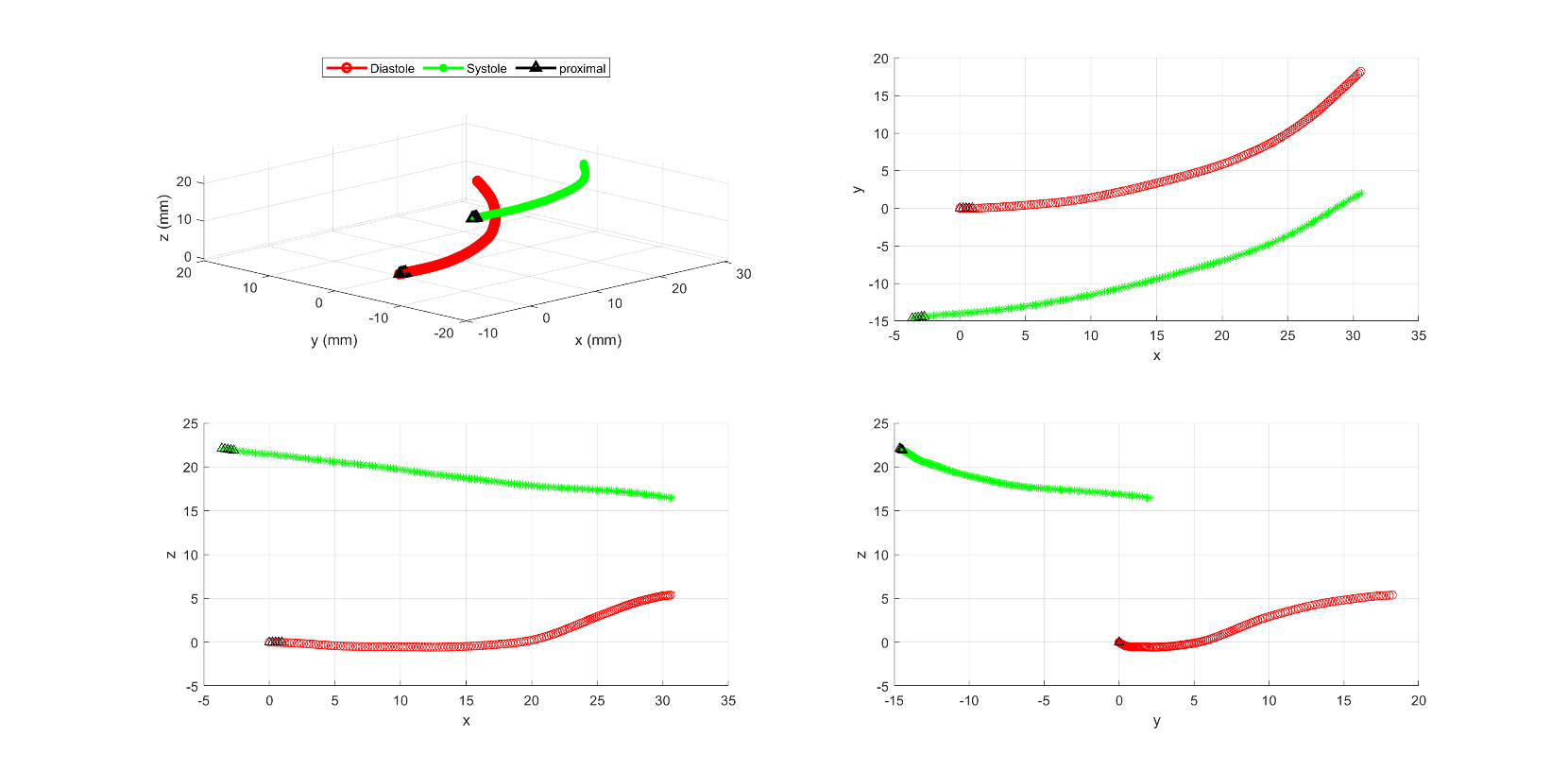


**RCA**

**Supplementary Figure 4**: Plots of showing multiple projections of vessel-centreline reconstructions used for calculation of bending displacement vectors, for LAD, LCx and RCA. Centrelines were extracted from vessel reconstructions based on sequential orthogonal angiograms of uninstrumented vessels, at diastole and systole.

**
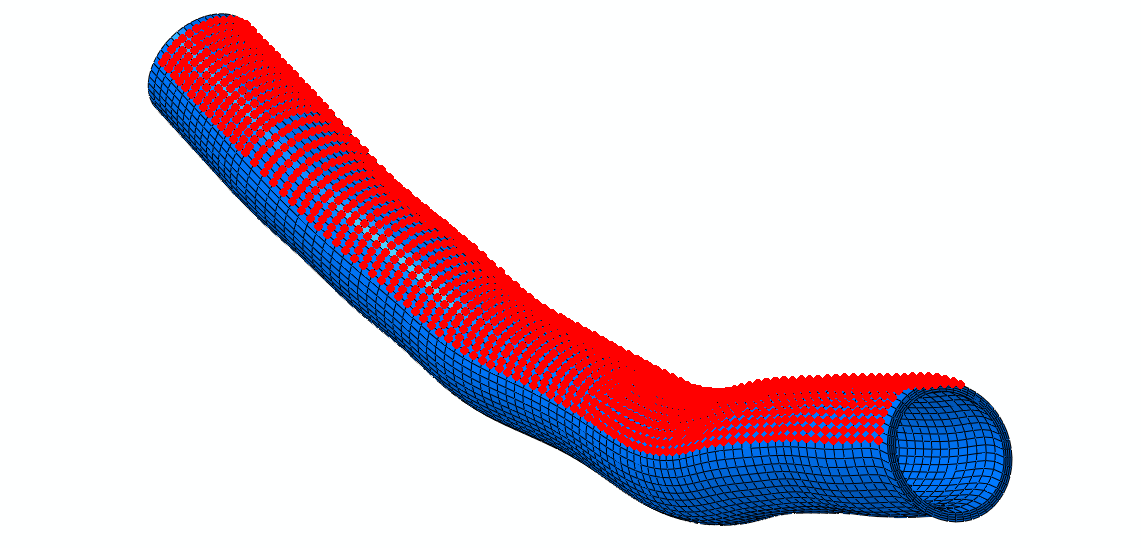
**

**Supplementary Figure 5**: Undeformed mesh of LCx vessel wall showing nodal locations of applied bending displacements in red. Bending displacement nodes are located on the inner curvature of the vessel and cover approximately 30% of the circumference. Inlet extension is shown. Inlet and outlet faces were also included in the bending displacements, but this was achieved via displacement of rigid contact planes.

**
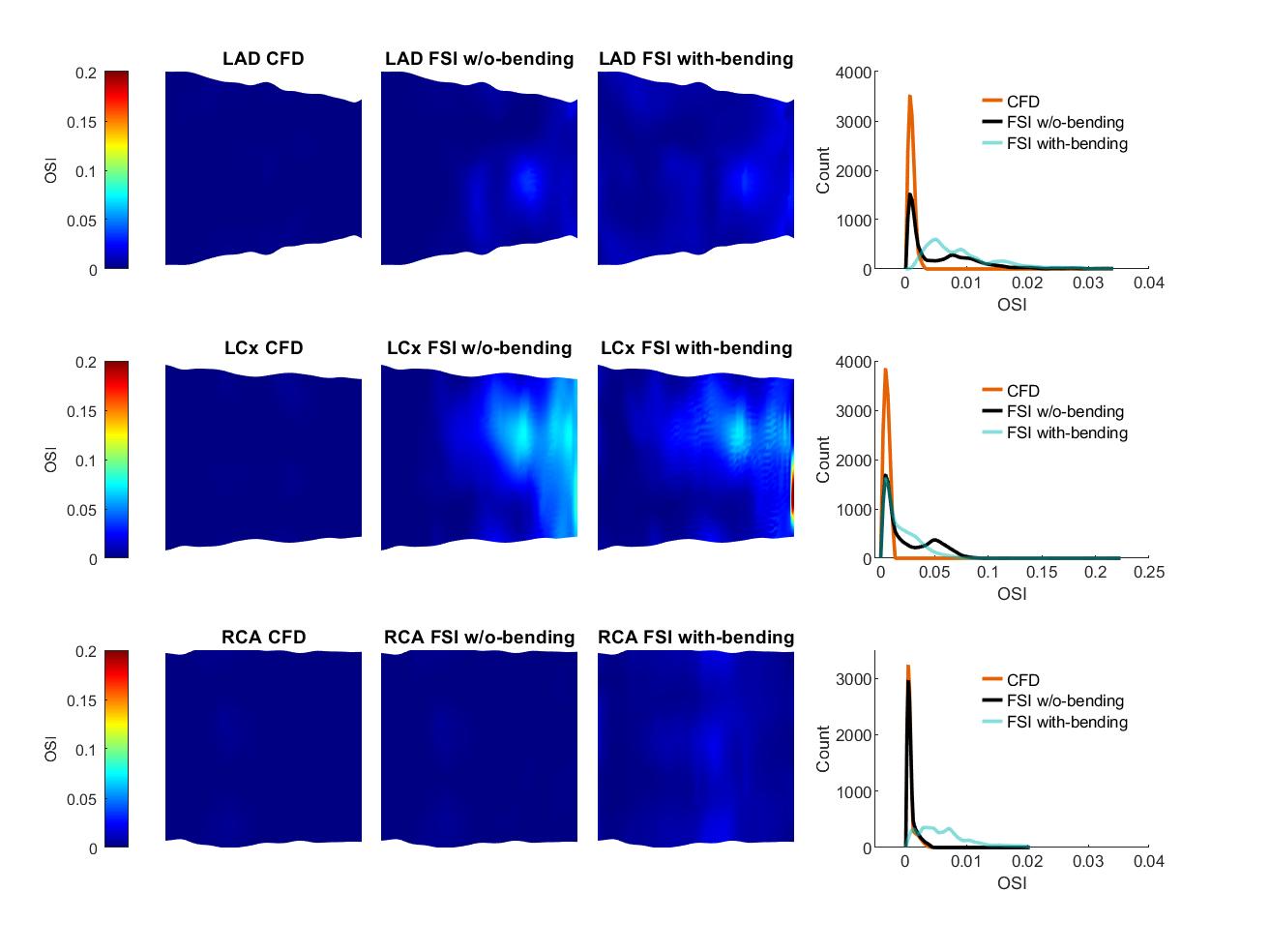
**

**Supplementary Figure 6**: Results of Oscillatory Shear Index (OSI) for rigid-wall CFD, FSI without (w/o) bending, and FSI with-bending, for LAD, LCx and RCA. Maps of each artery are presented as opened and flattened, showing magnitude and location of OSI for flow from proximal (left) to distal (right). Histograms compare the CFD, FSI without bending, and FSI with bending OSI datasets for each vessel.


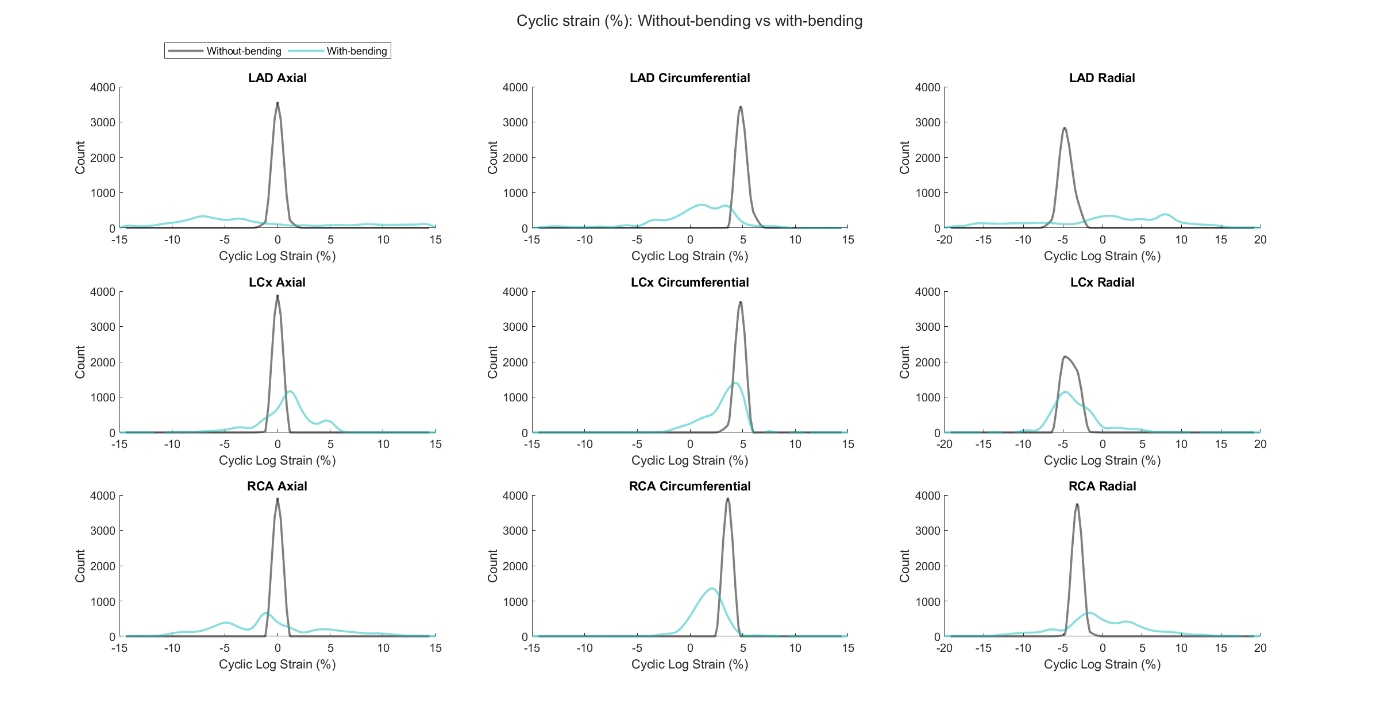


**Supplementary Figure 7**: Histograms comparing the three components of cyclic logarithmic strain (%) between FSI without bending and FSI with-bending, for LAD, LCx and RCA.
